# Multifactoriality of Parkinson’s Disease as Explored Through Human Neural Stem Cells and Their Transplantation in Middle-Aged Parkinsonian Mice

**DOI:** 10.3389/fphar.2021.773925

**Published:** 2022-01-19

**Authors:** Anna Nelke, Silvia García-López, Alberto Martínez-Serrano, Marta P. Pereira

**Affiliations:** ^1^ Tissue and Organ Homeostasis Program, Centro de Biología Molecular Severo Ochoa UAM-CSIC, Madrid, Spain; ^2^ Department of Molecular Biology, Faculty of Science, Universidad Autónoma de Madrid, Madrid, Spain

**Keywords:** aging, behavior, cell replacement therapy, neural stem cell, neuroinflammation, next-generation sequencing, parkinson’s disease, proteomics

## Abstract

Parkinson’s disease (PD) is an age-associated neurodegenerative disorder for which there is currently no cure. Cell replacement therapy is a potential treatment for PD; however, this therapy has more clinically beneficial outcomes in younger patients with less advanced PD. In this study, hVM1 clone 32 cells, a line of human neural stem cells, were characterized and subsequently transplanted in middle-aged Parkinsonian mice in order to examine cell replacement therapy as a treatment for PD. *In vitro* analyses revealed that these cells express standard dopamine-centered markers as well as others associated with mitochondrial and peroxisome function, as well as glucose and lipid metabolism. Four months after the transplantation of the hVM1 clone 32 cells, striatal expression of tyrosine hydroxylase was minimally reduced in all Parkinsonian mice but that of dopamine transporter was decreased to a greater extent in buffer compared to cell-treated mice. Behavioral tests showed marked differences between experimental groups, and cell transplant improved hyperactivity and gait alterations, while in the striatum, astroglial populations were increased in all groups due to age and a higher amount of microglia were found in Parkinsonian mice. In the motor cortex, nonphosphorylated neurofilament heavy was increased in all Parkinsonian mice. Overall, these findings demonstrate that hVM1 clone 32 cell transplant prevented motor and non-motor impairments and that PD is a complex disorder with many influencing factors, thus reinforcing the idea of novel targets for PD treatment that tend to be focused on dopamine and nigrostriatal damage.

## Introduction

Parkinson’s disease is the second most common neurodegenerative disease in the world and the most common movement disorder for which there is presently no cure. It is characterized by the death or impairment of dopaminergic neurons (DAn) in the substantia nigra pars compacta (SNpc) and the depletion of dopamine (DA) in the striatum (Str). It is this pathology that generates the motor symptoms, such as tremors, rigidity, bradykinesia, and postural instability, that are observed in PD patients (Lesage and Brice, 2009; Wirdefeldt et al., 2011; Kalia and Lang, 2015; De Virgilio et al., 2016).

Cell replacement therapy (CRT) is a promising approach to treating PD and is one of the few potential treatments that seek to replace or repair the DAn lost or damaged, respectively, in PD. Various cell sources such as human induced pluripotent stem cells, human mesenchymal stem cells and human neural stem cells (hNSCs), have been and are being used in clinical trials and in experimental PD models with varying degrees of success. Some of the beneficial effects of CRT observed are motor improvement many years post-transplant and surviving tyrosine hydroxylase (TH)+ grafted cells in the PD patients’ brain post-mortem (Hallett et al., 2014; Li et al., 2016; Stoker and Barker, 2016; Yasuhara et al., 2017; Stoker et al., 2018; U.S. National Library of Medicine, 2021).

One hNSC line that has thus far only been used in experimental PD models is the hVM1 clone 32 cell line. Upon differentiation *in vitro*, these fetal-derived hNSCs generate approximately 10% TH+ cells and around 12.5% β-III tubulin (TUBB3)+ cells. In addition, improvement of behavioral symptoms is observed in Parkinsonian rats when transplanted with hVM1 clone 32 cells (Ramos-Moreno et al., 2012b). When transplanted in 1-methyl-4-phenyl-1,2,3,6-tetrahydropyridine (MPTP)-intoxicated adult mice, hVM1 clone 32 cell transplant rescued striatal terminals and nigral neurons, reduced hyperactivity in mice, triggered mast cell migration to the superficial cervical lymph nodes, and restored neurogenesis and decreased microglial inflammation in the hippocampus (unpublished data).

Parkinson’s disease is an age-associated disorder, as age is the biggest risk factor for the development of the disease. Very few people are diagnosed with PD before the age of 40 and it is usually diagnosed after the age of 60 (Lesage and Brice, 2009; Wirdefeldt et al., 2011; Kalia and Lang, 2015; De Virgilio et al., 2016). Cell replacement therapy is more successful in younger patients with less advanced PD (Stoker and Barker, 2016; Yasuhara et al., 2017). Moreover, most *in vivo* PD transplantation studies using rodents and non-human primates use adult animals, omit age specifications, and/or do not correctly categorize the animals’ age group (Chaturvedi et al., 2006; Redmond et al., 2007; Gu et al., 2009; Kriks et al., 2011; Gonzalez et al., 2015; Jackson et al., 2017). By definition, an adult mouse or rat is between two and 3 months old. Considering the average lifespan of a mouse is 2 years and that of a rat is 3 years, the rodents used in many CRT studies extrapolate to a fairly young age in humans, much younger than 60 years old (Sengupta, 2013; Dutta and Sengupta, 2016). Therefore, although there is no doubt that progress has been made and that CRT has shown potential as a treatment for PD, it is important to have more *in vivo* studies done in older rodents in order to study what mechanisms are different from those in younger animals which lead to unsuccessful CRT results in older PD patients. Mice aged 10–15 months old are considered middle-aged and aged mice must be at least 18 months old (Jin et al., 2003; Luo et al., 2006; Dutta and Sengupta, 2016; Apple et al., 2017).

The aim of this study was to further characterize hVM1 clone 32 cells by means of gene and protein expression analyses, and to increase knowledge of CRT for PD in older rodents. To do so, hVM1 clone 32 cells were transplanted in middle-aged Parkinsonian mice, and 4 months later, the nigrostriatal pathway and behavior were analyzed to see if hNSC grafting would lead to an amelioration of these two important aspects in PD pathology.

## Materials and Methods

### Cell Culture

Use of hNSCs was approved by and adhered to the guidelines of the Research Ethics Committees of the Universidad Autónoma de Madrid and the Comunidad de Madrid (PROEX149/15). Details about the hVM1 clone 32 cells used in this study, including their human fetal origin as well as consent and donors, can be found in previous articles describing these cells (Villa et al., 2009; Ramos-Moreno et al., 2012b). Briefly, it is a clone isolated based on increased TH (DAn marker) generation from the stable, *v-myc*-immortalized hVM1 cell line. The hVM1 cells were generated from dissociated tissue of the ventral mesencephalon of a 10 week-old aborted human fetus. The hVM1 clone 32 cell line is a unique biological material developed by the Martínez-Serrano laboratory in 2009 and was authenticated by short tandem repeat profiling.

Cells were routinely cultured on plastic plates treated with 10 μg/ml polylysine (Sigma-Aldrich P1274) in proliferation medium. The proliferation medium composition was as follows: The base was Dulbecco’s modified Eagle’s medium/F-12, GlutaMAX supplement medium (Gibco 31331028), 1% AlbuMAX (Gibco 11020021), 50 mM HEPES (Gibco 15630106), and 0.6% D-glucose (Merck 104074). To this, 1X N2 supplement (Gibco 17502048), 1X homemade non-essential amino acids (composed of L-alanine, L-asparagine, L-aspartic acid, L-glutamic acid, and L-proline), 100 U/ml penicillin, 0.1 mg/ml streptomycin, 20 ng/ml human recombinant fibroblast growth factor 2 (R&D systems 233-FB), and 20 ng/ml human recombinant epidermal growth factor (R&D systems 236-EG), were added. Cells were grown at 37°C, in a 95% humidity, 5% CO_2_, and 5% O_2_ atmosphere. For differentiation experiments, multiwell plates were treated with 30 μg/ml polylysine overnight, and then incubated with laminin at 5 μg/ml (Sigma-Aldrich L2020) for 5 h, before seeding cells into wells. Cells were seeded at 20,000 cells/cm^2^, in proliferation medium. Twenty-four hours later, this medium was replaced with differentiation medium, which is the same one used for proliferation experiments, but the growth factors are replaced with 2 ng/ml human recombinant glial cell-derived neurotrophic factor (GDNF) (Peprotech 450-10) and 1 mM dibutyryl cAMP (Sigma-Aldrich D0627) (Lotharius et al., 2002). One day later, the differentiation medium was fully changed, and after this, two thirds of the differentiation medium were changed every 2 days. Differentiated cell samples were collected after 7 days of differentiation. Equivalent multiwell plates with proliferation medium were seeded in parallel and these samples were collected at 3 days post-seeding.

### Next-Generation Sequencing

An exploratory NGS study was performed using proliferating and differentiated hVM1 clone 32 cells in order to analyze their differential expression in both of these conditions. Cell culture media was removed and cells were rinsed with 1X PBS. In order to isolate RNA from the samples, TRIzol Reagent (Invitrogen 15596026) was added to the cells and RNA extraction was done using the Direct-zol RNA Miniprep Plus kit (Zymo Research R2071). Whole-transcriptome analysis was done on RNA samples with Illumina total RNA-Seq technology using the ScriptSeq Complete kit (Illumina RS-122-2201), which entailed rRNA removal, cDNA synthesis, 3′ terminal tagging, and PCR purification, followed by sequencing using the NextSeq 550 Sequencing kit and system (Illumina). Reads were generated from raw total RNA-Seq data and mapped to the human genome, and the htseq-count tool was used to count the number of reads mapping each gene. Differential expression analysis was then performed on raw total RNA-Seq data using the DESeq2 package, with differences in gene expression between dividing and differentiated cells being expressed as fold change. Negative fold change indicates that the gene is more highly expressed in proliferating cells, and positive fold change indicates that the gene is more highly expressed in differentiated cells. All NGS datasets can be found online at https://doi.org/10.21950/4IXBTX.

### Proteomic Analysis

A proteomic study was employed in order to analyze the differential protein expression of the hVM1 clone 32 cells in proliferation and differentiation conditions. Cells were washed with 1X PBS and dissociated with accutase (Merck SCR005). The 2002 base medium without 1% AlbuMAX was then added to the plates and the cells were centrifuged at 2,500 rpm at RT for 10 min. The supernatant was removed, and pellets were collected and stored at −80°C. Pellets were lysed using lysis buffer containing 7 M urea, 2 M thiourea, 100 mM triethylammonium bicarbonate, 5% sodium dodecyl sulfate, and a nuclease. Total protein concentration was quantified using the Pierce 660 nm protein assay (ThermoFisher Scientific). Reduction of disulfide bonds and subsequent alkylation was then carried out using 50 mM tris(2-carboxyethyl)phosphine and 200 mM methyl methanethiosulfonate, respectively. After this, protein digestion was performed using S-Trap columns (Protifi) according to the manufacturer’s instructions. In brief, 20 μg of each sample was digested overnight at 37°C with trypsin at a 20:1 protein to enzyme ratio. The digested samples were then dried in a SpeedVac vacuum concentrator (ThermoFisher), and peptide concentration was quantified using a fluorometer (QuBit). Next, high resolution label free quantitation liquid chromatography-electrospray ionization-tandem mass spectrometry, with the liquid chromatography system and mass spectrometer connected, was performed. To do so, 1 μg of each sample underwent liquid chromatography using a C18 reversed phase column in order to separate the peptides by their polarity, then the eluted peptides were separated using a TripleTOF mass spectrometer (Sciex). Mass spectrometry data was analyzed employing four different database searches, namely Mascot Server version 2.5 (Matrix Science), OMSSA version 2.1.9 (NCBI), X! TANDEM version win-13-02-01-1 (The GPM), and Myrimatch version 2.1 (Vanderbilt University), against the Uniprot *Homo sapiens* database (78,120 proteins when last updated on 2021/03/07). False discovery rate for peptides was < 1% (*q* < 0.01) and < 5% (*q* < 0.05) for proteins. Peptide mass tolerance was set to 0.05 Da and allowed missed cleavages was 2. L-cysteine methyl disulfide was a fixed peptide modification, and the variable modifications were methionine oxidation, pyrogluatmic acid from glutamine, pyroglutamic acid from glutamic acid, and acetylation of protein B-terminus, the latter of which was a post-translational modification. Differential expression of proliferating and differentiated hVM1 clone 32 cells was expressed as fold change. Negative fold change indicates that the gene is more highly expressed in proliferating cells, and positive fold change indicates that the gene is more highly expressed in differentiated cells. The Qiagen Ingenuity Pathway Analysis was then used for functional analysis including canonical pathways, diseases and disorders, biological functions, networks, and top upstream regulators. Because of the large amount of proteins with *q* < 0.05, log2 (Fold change) threshold was set to 0.58 (*q* < 0.01) for these functional analyses in order to increase rigor and only include proteins with the biggest fold change. For canonical pathways and top upstream regulators, z-score threshold was set to ≥1. Inflammatory response was considered a biological function, and not a disease or disorder. Chemicals were omitted and only proteins were reported as top upstream regulators. All proteomic datasets, including all information used to identify peptides and proteins, can be found online at https://doi.org/10.21950/06EW6H.

### Immunocytochemistry

For immunocytochemistry studies, cells were seeded on cover glasses in multiwall plates. Cells were fixed with cold 4% paraformaldehyde for 15 min and stored in cryoprotectant solution (30% glycerol and 30% ethylene glycol in 1X PBS). Samples were washed in 1X TBS, blocked in 10% serum in 1X TBS/0.5% TritonX-100, and incubated overnight at 4°C with primary antibodies in 2% serum in 1X TBS/0.5% TritonX-100. The following primary antibodies were used: TH (1:250; Sigma-Aldrich T1299), TUBB3 (1:250; Sigma-Aldrich T2200), Ki-67 (1:200; ThermoFisher Scientific RM-9106-S1), vimentin (VIM) (1:500; Santa Cruz sc-6260), microtubule-associated protein 2 (MAP2) (1:250; Sigma-Aldrich M4403), synapsin I (SYN1) (1:250; Merck AB1543), glial fibrillary acidic protein (GFAP) (1:500; DAKO Z0334), and γ-aminobutyric acid (GABA) (1:1,000; Sigma-Aldrich A2052). The samples were then washed and incubated with appropriate secondary antibodies in 2% serum in 1X TBS/0.5% TritonX-100, at RT for 2 h. The following secondary antibodies were used: Biotinylated Horse Anti-Mouse IgG Antibody, rat adsorbed (1:500; Vector Laboratories BA-2001), Goat anti-Rabbit IgG (H + L) Highly Cross-Adsorbed Secondary Antibody, Alexa Fluor 647 (1:500; Invitrogen A-21245), Goat anti-Rabbit IgG (H + L) Highly Cross-Adsorbed Secondary Antibody, Alexa Fluor 546 (1:500; Invitrogen A-11035), and Goat anti-Mouse IgG (H + L) Highly Cross-Adsorbed Secondary Antibody, Alexa Fluor 546 (1:500; Invitrogen A-11030). For TH and MAP2 immunostainings, samples were subsequently washed and incubated with streptavidin (Invitrogen SA1010) in 1X TBS/0.5% TritonX-100 at RT for 45 min. Nuclei were stained with 4′,6-Diamidino-2-phenylindole dihydrochloride (DAPI; 1:1,000; Santa Cruz sc-3598). All samples were then washed with 1X TBS, air-dried, and mounted with homemade Mowiol mounting medium, composed of 10% MOWIOL 4-88 Reagent (Merck 475904).

### Animal Procedures

All animal work was approved by and adhered to the guidelines of the Research Ethics Committees of the Universidad Autónoma de Madrid and the Comunidad de Madrid (PROEX149/15). Animal procedures were performed at the Animal Facility of the Centro de Biología Molecular Severo Ochoa. All animal experiments complied with the ARRIVE guidelines and were carried out in accordance with the EU Directive 2010/63/EU guidelines. All efforts were made to minimize animal suffering and to reduce the number of animals used.

Male C57BL/6JRccHsd mice (Envigo, Netherlands) were used in this study. Animals were 12 months old with an average weight of 40.5 g at the beginning of the experiment, and were housed in a temperature- and humidity-controlled room on a 12-h light/dark cycle, and fed *ad libitium* with standard food and water. Animals were randomly separated into one of three experimental groups: Control, MPTP + buffer, and MPTP + cell. Control mice were injected i.p., with 0.9% saline once every 2 hours, with a total of three injections, at 10 μl/g. Using the same injection protocol, PD was induced in other mice by injecting MPTP (Sigma-Aldrich M0896) i.p., at 15 mg/kg. The dose of 15 mg/kg was chosen in order to have intermediary nigrostriatal damage before the transplant since it has been shown that when MPTP is administered at 18 mg/kg or 20 mg/kg once every 2 hours, with a total of four injections, there is a major loss of TH+ fibers and cells in the Str and SNpc, respectively (Jackson-Lewis and Przedborski, 2007). During pilot studies, a very high percentage of mice died after the fourth injection and therefore, it was decided that only three injections would be made. Mortality rate was 23% following MPTP injections. One month later, mice injected with MPTP underwent stereotaxic surgery to receive an intracerebral injection in the left Str (Coordinates from Bregma: Anteroposterior 0.25 mm, Mediolateral 2.75 mm, Dorsoventral 3 mm) of either 1.5 μl transplantation medium (MPTP + buffer group) or 100,000 mycoplasm-free, undifferentiated hVM1 clone 32 cells in passage 26 in 1.5 μl transplantation medium (MPTP + cell group). The timepoint of 1 month was used to create a more translational model, transplantation not immediately after the start of PD pathology and later than 1 week post-MPTP injections because the loss of DAn in the SNpc is stable by this time (Jackson-Lewis and Przedborski, 2007). The transplantation medium was composed of the following: 49% Leibovitz’s L-15 Medium (ThermoFisher Scientific 11415064), 49% filtered 0.6% Glucose (Merck 104074) in 1X PBS, and 2% B-27 Serum-Free Supplement (Gibco 17504044). The transplantation medium is different than the proliferation medium and contains Leibovitz’s L-15 Medium so that the hVM1 clone 32 cells are nourished while transitioning from *in vitro* to *in vivo* in an unstable environment in terms of temperature as well as O_2_ and CO_2_ levels, and to make sure that these hNSCs are not dividing which could cause tumor growth upon transplantation into the mouse brain.

For surgery, animals were anesthetized with a mixture of ketamine at 80 mg/kg (Merial) and xylazine at 10 mg/kg (Calier) injected i.p., When animals were confirmed to be asleep *via* toe pinching, surgery began. After positioning the animal’s head in the frame of the stereotaxic apparatus, the animal’s skull was revealed and a 23-gauge needle with 0.635 mm outer diameter was used to make a hole in the skull. Through this hole, a 22-gauge needle (Hamilton Company; 22 gauge, Small Hub RN NDL, length 0.75 in, point style 4 cut at an angle of 10–12°) held in the attached syringe (Hamilton Company; 10 μl, Model 701 RN, 26s gauge, 51 mm, point style 2), was lowered into the brain to inject either the transplantation medium or hNSCs in the left Str. The speed of injection was 1 μl/min, and the needle was left in for 2 min after injecting cells before its slow removal. The antibiotic oxytetracycline (0.2 mg/ml; Terramycin^®^, Zoetis) was delivered *ad libitium* in drinking water of MPTP-treated animals starting the day of surgery for a total period of 1 week as a preventative measure. In order to avoid graft rejection, 2 days before the transplants and for the first week post-transplant, all animals were given an i.p. injection of cyclosporine A (CSA; Novartis) at 10 mg/kg once daily. For the remainder of the experiment, all animals were treated with daily weekday i.p., injections of CSA at 10 mg/kg, and twice a week, CSA was included in the drinking water, prepared with the following components: 0.25 g/L CSA and sweetener. The immunosuppression protocol was based on previous studies (Courtois et al., 2010; Ramos-Moreno et al., 2012a; Ramos-Moreno et al., 2012b). For the twice weekly inclusion of CSA in drinking water, oral administration has been shown to be effective in combination with injections (Jensen et al., 2012). Preparation of oral CSA was based on the average daily water intake by C57BL mice which is 4 ml (Tordoff et al., 2007) and body weight (approximately 40 g), which amounted to 0.25 g/L CSA in water. Four months post-transplant, all animals were sacrificed.

### Behavioral Tests

In order to detect neurological and motor alterations, animals were subjected to the open field test (OFT) and paw print test (PPT) (Prut and Belzung, 2003; Formentini et al., 2014). Animals received three training sessions prior to taking basal measurements for all behavioral tests. All OFTs were performed in the same room, with the same lighting, and at the same time.

#### Open Field Test

Animals were placed in a 40 cm × 40 cm × 30 cm (L × W × H) four-walled cubic box and their movements were filmed for 10 min. Time spent in the center (20 cm × 20 cm central area), distance traveled, time spent grooming (mouse licks or scratches itself while stationary), time spent rearing (mouse stands on hind legs), urination (number of puddles or streaks of urine), and defecation (number of fecal boli), were measured using the ANY-maze behavioral tracking software.

#### Paw Print Test

The animals’ paws were painted (forelimbs in green and hindlimbs in orange) and the mice then walked on a 40 cm × 12 cm white piece of paper. Contralateral (CL) and ipsilateral (IL) stride length (the distance between two same-sided forelimbs or two same-sided hindlimbs), and CL-IL and IL-CL stride width (the distance between two opposite-sided forelimbs or two opposite-sided hindlimbs), were measured.

### Immunohistochemistry

Transcardial perfusion fixation was carried out using cold 4% paraformaldehyde. After 12-h post-fixing with 4% paraformaldehyde, the tissue was dehydrated in 30% sucrose until the tissue sank. Free-floating 15 μm-thick coronal sections of the brain were sliced using a freezing microtome and then stored in cryoprotectant solution at −20°C.

Brain sections were washed and then blocked in 3–5% serum in 1X PBS/0.3% TritonX-100 and incubated overnight with primary antibody in 1% serum in 1X PBS/0.3% TritonX-100 at 4°C. The following primary antibodies were used: STEM121 (1:500; Takara Bio Y40410), GFAP (1:1,000; DAKO Z0334), TH (1:400; Pel-Freez P40101-150), dopamine transporter (DAT) (1:400; Chemicon MAB369), Iba1 (1:1,500; Wako 019-19741), and nonphosphorylated neurofilament heavy (NFH) (1:500; Biolegend 801701).

For the TH immunostaining, sections were incubated with a mix of 1% of 30% hydrogen peroxide, 3% methanol, and 6% 1X PBS for 15 min, prior to blocking. After primary antibody incubation, TH-stained sections were incubated with biotinylated secondary antibody (Biotinylated Goat Anti-Rabbit IgG Antibody; 1:500; Vector Laboratories BA-1000) in 1% serum in 1X PBS/0.3% TritonX-100, washed, incubated in ABC solution (VECTASTAIN Elite ABC HRP Kit, Vector Laboratories PK-6100), washed, and developed with the Vector VIP Peroxidase (HRP) Substrate kit (Vector Laboratories SK-4600). Samples were mounted onto glass slides (Menzel-Gläser), air-dried, dehydrated with xylene, and coverslipped with distyrene, plasticiser, and xylene mounting medium.

For fluorescent immunohistochemistry samples, after primary antibody incubation, sections were washed and incubated with adequate secondary antibodies in 1% serum in 1X PBS/0.3% TritonX-100 at RT for 2 h. The following secondary antibodies were used: Goat anti-Mouse IgG (H + L) Highly Cross-Adsorbed Secondary Antibody, Alexa Fluor 546 (1:1,000; Invitrogen A-11030), Goat anti-Rabbit IgG (H + L) Highly Cross-Adsorbed Secondary Antibody, Alexa Fluor 647 (1:1,000; Invitrogen A-21245), Goat anti-Rat IgG (H + L) Cross-Adsorbed Secondary Antibody, Alexa Fluor 555 (1:500; Invitrogen A-21434), Goat anti-Rabbit IgG (H + L) Highly Cross-Adsorbed Secondary Antibody, Alexa Fluor 488 (1:1,000; Invitrogen A-11034), and Biotinylated Horse Anti-Mouse IgG Antibody, rat adsorbed (1:500; Vector Laboratories BA-2001). Nonphosphorylated NFH sections were subsequently washed and incubated with streptavidin (Invitrogen SA1010) in 1X PBS/0.3% TritonX-100 at RT for 1 h. All fluorescent samples’ nuclei were stained with DAPI (1:1,000; Santa Cruz sc-3598). Sections were washed, mounted onto glass slides (Menzel-Gläser), air-dried, and coverslipped with homemade Mowiol mounting medium, composed of 10% MOWIOL 4-88 Reagent (Merck 475904).

### Histological Quantifications

Microscope images were obtained for all immunostainings. Anteroposterior coordinate ranges from Bregma for the quantified brain sections were 0.98–0.38 mm for the Str and motor cortex, and −2.80 to −3.64 mm for the SNpc (Paxinos and Franklin, 2001). For TH, DAT, GFAP, and NFH, immunostainings, region of interest was drawn by selecting anatomical borders, threshold was set to be the same for all animals for each immunohistochemistry, and area fraction was measured using a custom macro to analyze the images semi-automatically. The TH area fraction in the Str was made up of fibers and that of the SNpc was made up of cells and their prolongations. ImageJ was used to do all quantifications.

### Statistical Analysis

**FIGURE 1:**
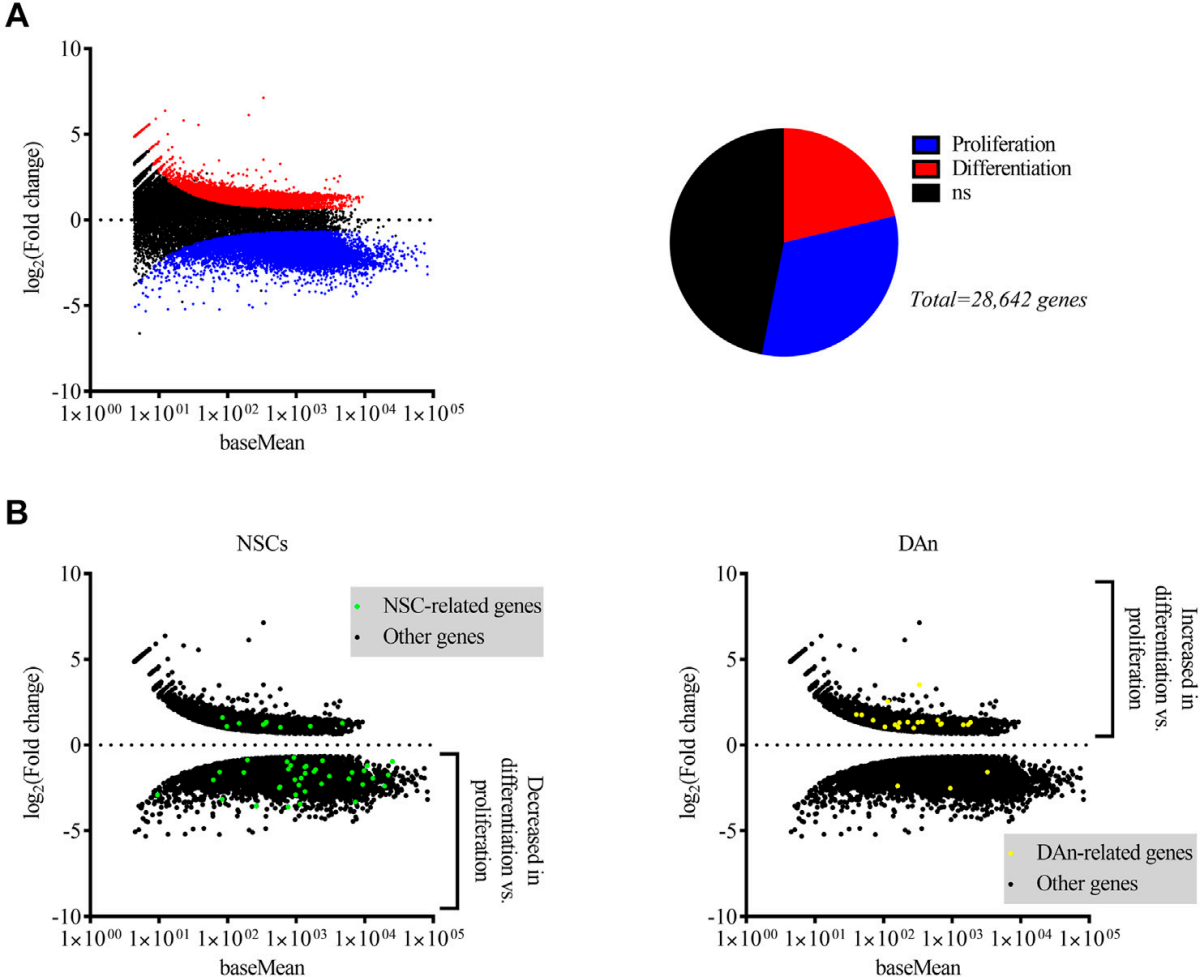
Differential expression of genes by dividing and differentiated hVM1 clone 32 cells indicated upregulation of NSC-associated genes in proliferation and of DAn-associated genes when differentiated. **(A)** MA plot **(left)** and pie chart **(right)** showing genes differentially expressed by hVM1 clone 32 cells in proliferation (blue dots and slice) and differentiation (red dots and slice) conditions. Black dots and slice indicate genes that had a non-significant q value (0.05 < *q* < 1). **(B)** MA plots representing NSC- (**left**; green dots) and DAn-related (**right**; yellow dots) differential gene expression. Black dots indicate differentially expressed genes not associated with either NSCs or DAn. NSC-related genes were increased in proliferation, while those related with DAn were increased in differentiation. All MA plots show the mean of normalized counts on the *x*-axis and log2 fold changes on the *y*-axis. All significantly differentially expressed genes had *q* < 0.05. Proliferation *n* = 2, Differentiation *n* = 2. Data were obtained from two independent experiments.

**FIGURE 2:**
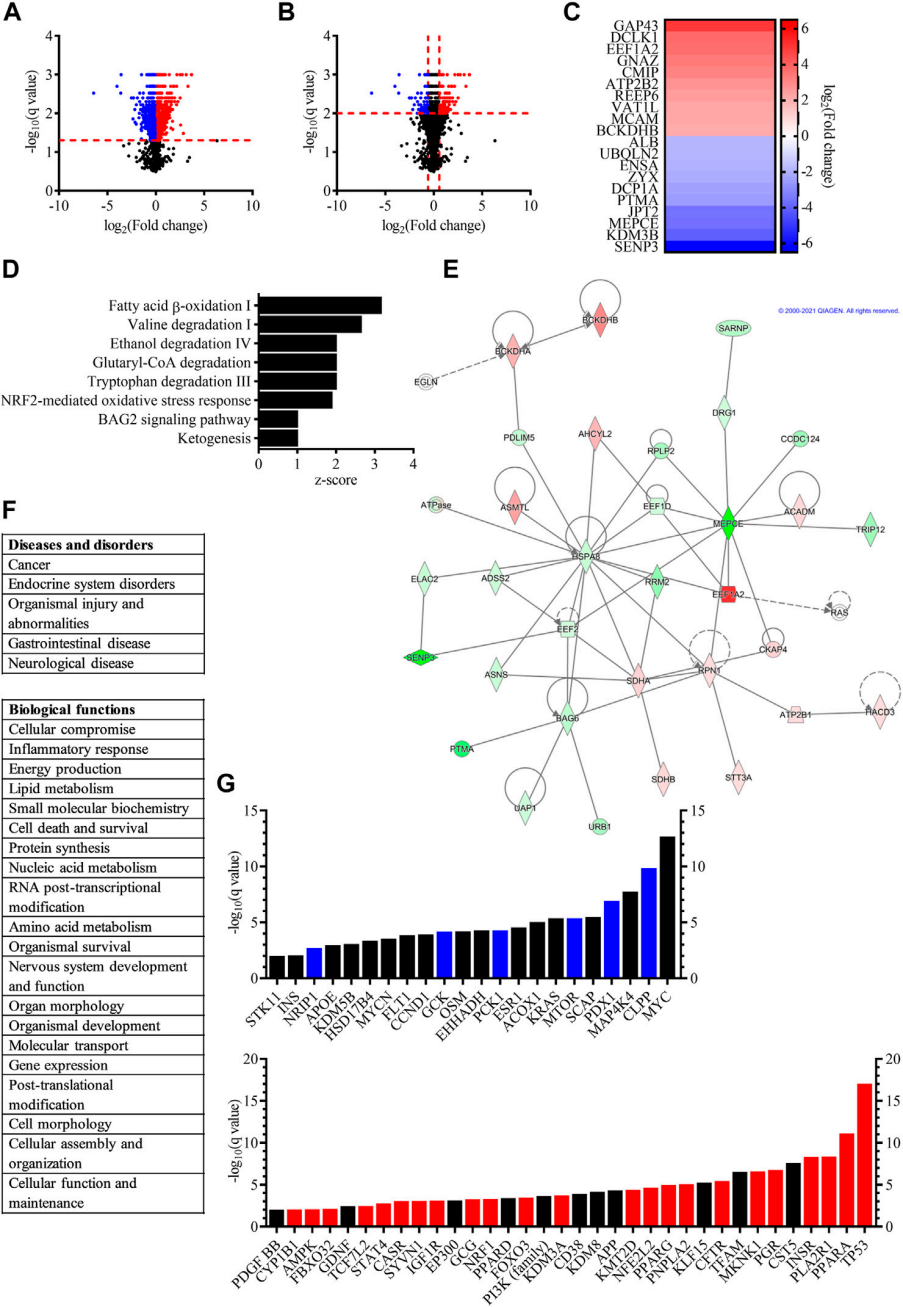
Proteomic study revealed that proliferating and differentiated hVM1 clone 32 cells are influenced by a wide variety of proteins and pathways. **(A)** Volcano plot, with fold change on the *x*-axis and the q value on the *y*-axis, showing differentially expressed proteins in dividing (blue dots) and differentiated (red dots) hNSCs with *q* < 0.05. Black dots indicate proteins that had a non-significant *q* value. **(B)** Volcano plot, with fold change on the *x*-axis and the *q* value on the *y*-axis, demonstrating differentially expressed proteins in proliferating (blue dots) and differentiated (red dots) hNSCs with *q* < 0.01. Black dots indicate proteins that had *q* > 0.01. **(C)** Heat map illustrating the proteins with the biggest fold change increased in dividing (blue) and differentiated (red) hVM1 clone 32 cells. **(D)** Bar graph showing the top canonical pathways of proliferating and differentiated hNSCs, with bars indicating the z-score of each pathway. **(E)** One of the top networks relevant to hVM1 clone 32 cells. Image from QIAGEN Ingenuity Pathway Analysis. **(F)** List of top diseases and disorders **(top)**, and list of top biological functions **(bottom)** of hVM1 clone 32 cells. **(G)** Bar graph illustrating the top upstream regulators of dividing **(top)** and differentiated **(bottom)** hNSCs, with bars indicating the q value. Blue and red bars indicate proteins that were activated in proliferating and differentiated cells, respectively. Black bars represent proteins that were top upstream regulators, but not activated. For panels **(D–G)**, the *q* value was set to 0.01. Proliferation *n* = 5, Differentiation *n* = 5. All data were obtained from at least three independent experiments.

For NGS, three proliferation and three differentiation individual samples were obtained from three independent experiments, however one sample from each group had to be excluded upon analysis because they were identified as outliers likely due to distinct DNA extraction method. Five samples of each experimental condition from at least three individual experiments were analyzed in the proteomic study. The animal experiments and immunostainings were done once, with a total of 30 animals used. A minimum of three animals were used per experimental group. Exact n used for each experiment is indicated in the figure legends. All figures were made and all statistical analyses were done using GraphPad Prism 7. The MA plots in Figure 1 show the log2 fold changes between two conditions over the mean of normalized counts for all samples. Figure 1A, right is a pie chart with slices representing differentially expressed genes in proliferation (blue), differentiation (red), and those that were not statistically significant (black). The volcano plots in Figures 2A,B illustrate the fold change on the *x*-axis and the q value on the *y*-axis. Figure 2C is a heat map showing proteins with the largest fold changes in proliferation (blue) and differentiation (red) conditions, Figure 2D is a bar graph with bars indicating the z-score for each canonical pathway, and Figure 2E is an image produced by the QIAGEN Ingenuity Pathway Analysis of one of the top networks. In Figure 2G, bars indicate the q value of each top upstream regulator, which are in black, except for those that are activated in dividing and differentiated hNSCs, which are blue and red, respectively. In Figures 4–6, graph columns represent mean values and error bars indicate standard error of the mean. For comparisons between more than two groups, the Brown-Forsythe test was used to test the homogeneity of variances. Standard deviations were significantly different only in the case of nonphosphorylated NFH expression in the motor cortex, but the result was not affected as changes between groups were not significant in all cases. Furthermore, the Shapiro-Wilk normality test was used to test the normality of populations. When this normality test was passed or when this normality test was not passed but the Kolmogorov-Smirnov normality test was passed, a one-way analysis of variance (ANOVA) followed by Tukey’s multiple comparisons post-hoc test were performed in order to compare the mean of each column with the mean of every other column. When neither normality test was passed, the Kruskal-Wallis test followed by Dunn’s multiple comparisons post-hoc test were performed in order to compare the mean of each column with the mean of every other column. For NGS and proteomic experiments, a *q* value of less than 0.05 was considered statistically significant, except for proteomic functional analyses where a *q* value of less than 0.01 was considered significant. For immunohistochemistry and behavior experiments, a *p* value of less than 0.05 was considered statistically significant.

## Results

### Dividing hVM1 Clone 32 Cells Expressed Neural Stem Cell-Associated Genes and When Differentiated, These Cells Expressed Genes Essential for Dopaminergic Neurons

A NGS study was performed on RNA extracted from the hVM1 clone 32 cells in order to do a differential gene expression analysis between proliferating and differentiated hNSCs, allowing us to identify what genes were expressed in both groups and if there was an increase or decrease in the expression of genes found in both groups. Around 34,000 genes were matched to the human genome in proliferating cells and more than 37,000 genes in differentiated cells were mapped to this genome. A total of 28,642 differentially expressed genes with a *q* value <1 were identified, of which 15,212 were statistically significant. Among these, 6,090 genes had a positive fold change, thus being more highly expressed in differentiated cells, and 9,122 gene had a negative fold change, indicating that they were more highly expressed in dividing cells (Figure 1A). The genes of higher interest were those related to NSCs and DAn. The majority of NSC-associated genes were upregulated in dividing cells and downregulated in differentiated cells, while the majority of DAn-associated genes were upregulated in differentiated cells and downregulated in proliferating cells (Figure 1B).

Hallmark NSC markers nestin (*NES*) and *VIM* were upregulated in proliferation conditions compared to differentiation, while surprisingly SRY-box transcription factor 2 (*SOX2*) was not detected in the whole-transcriptome analysis. Other NSC-associated genes with higher expression levels in proliferation compared to differentiation, were brain lipid-binding protein (*BLBP*), hes family bHLH transcription factor 1 (*HES1*), marker of proliferation Ki-67 (*MKI67*), notch receptor 1 (*NOTCH1*), noggin (*NOG*), and NUMB endocytic adaptor protein (*NUMB*). Immature neuron marker stathmin 1 (*STMN1*) had higher expression in division while neuroblast marker doublecortin (*DCX*) had higher expression in differentiation. Many genes important in the maintenance of mature neurons were upregulated in differentiated cells, most notably calbindin 2 (*CALB2*), neural cell adhesion molecule 2 (*NCAM2*), RNA binding protein fox-1 homolog 3 (*NEUN*), neuregulins, neurexins, synapsins including *SYN1* and synapsin II (*SYN2*), synaptotagmins, synaptopodins, synaptoporins, and microtubule associated protein Tau (*MAPT*) (Supplementary Tables S1, S2).

As for genes involved in DAn generation and maintenance, *DAT*, potassium inwardly rectifying channel subfamily J member 6 (*GIRK2*), LIM homeobox transcription factor 1 alpha and 1 beta (*LMX1A* and *LMX1B*), paired like homeodomain 3 (*PITX3*), and *TH*, among others, were highly expressed by hVM1 clone 32 cells after 7 days of differentiation. Three genes expressed by DAn, engrailed homeobox 1 and 2 (*EN1* and *EN2*), and orthodenticle homeobox 2 (*OTX2*), had increased expression in dividing hNSCs. Signaling by Wnt family member 2 and 3a (*WNT2* and *WNT3A*), as well as bone morphogenetic protein 2 and 5 (*BMP2* and *BMP5*), have been shown to be important in DAn differentiation (Sousa et al., 2010; Andersson et al., 2013; Liu et al., 2013; Jovanovic et al., 2018), and all of these genes were also positively differentially expressed by the differentiated hNSCs (Supplementary Table S1). Furthermore, expression of fibroblast growth factor 1, 2, 8, 9, and 20 (*FGF-1*, *FGF-2*, *FGF-8*, *FGF-9*, and *FGF-20*), all of which play a role in the survival, differentiation, and protection of DAn (Xia et al., 2016; Tome et al., 2017), were present in the differentiated hNSCs’ transcriptome (data not shown). Several neurotrophic factor (NTF) receptors involved in DAn survival were upregulated in differentiated cultures, namely genes coding for GDNF, artemin, and neurturin receptors GDNF family receptor alpha 1 and 2 (*GFRA1* and *GFRA2*), as well as neurotrophic receptor tyrosine kinase 1 (*NTRK1*) which binds nerve growth factor and neurotrophin 3, neurotrophic receptor tyrosine kinase 2 (*NTRK2*) which binds brain-derived neurotrophic factor (BDNF), in addition to neurotrophin 3 and 4, and neurotrophic receptor tyrosine kinase 3 (*NTRK3*), which binds neurotrophin 3 (Allen et al., 2013; Tome et al., 2017). Several vascular endothelial growth factor (VEGF) family members, which have been shown to be neuroprotective for DAn both *in vitro* and *in vivo* (Yasuhara et al., 2004; Falk et al., 2010; Piltonen et al., 2011), and their receptors were expressed in both proliferating and differentiated hVM1 clone 32 cells, including VEGF A, B, and C (*VEGFA*, *VEGFB*, and *VEGFC*), VEGF receptor 1 and 2 (*VEGFR1* and *VEGFR2*), and neuropilin 1 and 2 (*NRP1* and *NRP2*). Additionally, astrocyte-secreted NTFs mesencephalic astrocyte-derived neurotrophic factor (*MANF*) and ciliary neurotrophic factor (*CNTF*) were more highly expressed in proliferating cultures. Both of these NTFs promote DAn survival (Sullivan and Toulouse, 2011; Tome et al., 2017) (Supplementary Table S1).

Genes involved in central nervous system (CNS) immunity were also differentially expressed in proliferating and differentiated hVM1 clone 32 cells. The monocyte chemoattractant protein 1 (*MCP1*) and stem cell factor (*SCF*) genes were upregulated in proliferating cells, while their receptors, C-C motif chemokine receptor 2 (*CCR2*) and KIT proto-oncogene, receptor tyrosine kinase (*KIT*), respectively, were expressed higher under differentiation conditions. In a PD animal model, *SCF* was shown to have a protective effect on DAn (Yasuhara et al., 2006). Furthermore, genes encoding pro-inflammatory cytokines interleukin 1 beta and 6 (*IL1B* and *IL6*), as well as prostaglandin-endoperoxide synthase 2 (*PTGS2*) were upregulated in differentiated cells (Supplementary Table S1).

The hVM1 clone 32 cells also expressed astrocyte-related genes. These included *HES1*, S100 calcium binding protein B (*S100B*), and solute carrier family 1 member 3 (*SLC1A3*), which were more upregulated in dividing hNSCs, as well as aldehyde dehydrogenase 1 family member L1 (*ALDH1L1*) and *GFAP*, which were more highly expressed in the differentiated state. Moreover, oligodendrocyte-associated genes myelin basic protein (*MBP*), myelin-associated oligodendrocyte basic protein (*MOBP*), and oligodendrocytic myelin paranodal and inner loop protein (*OPALIN*), were expressed higher under differentiation conditions compared to proliferation conditions. Serotonergic neuron-related genes solute carrier family 6 member 4 (*SLC6A4*) and tryptophan hydroxylase 2 (*TPH2*), GABAergic neuron-related genes GABA type B receptor subunit 1 and 2 (*GABBR1* and *GABBR2*), and solute carrier family 6 member 1 (*SLC6A1*), glutamatergic neuron-associated genes glutamate ionotropic receptor NMDA type subunit 2B (*GRIN2B*) and solute carrier family 17 member 6 (*SLC17A6*), and cholinergic neuron-associated gene choline O-acetyltransferase (*CHAT*), all had increased expression in differentiated compared to dividing hNSC cultures (Supplementary Table S1). Other genes of interest found to be expressed higher in differentiated versus proliferating cultures were synuclein alpha (*SNCA*) and leucine rich repeat kinase 2 (*LRRK2*), two genes mutated in familial PD (Lesage and Brice, 2009; Chai and Lim, 2013) (Supplementary Table S2). More genes differentially expressed in proliferating and differentiated hVM1 clone 32 cells can be found in Supplementary Table S2.

### Proteomic Analyses of hVM1 Clone 32 Cells Demonstrated That Dopaminergic Neurons and Parkinson’s Disease are Multifaceted

A differential protein expression analysis between proliferating and differentiated hVM1 clone 32 cells, was performed in order to identify what proteins are expressed in both groups and if there was an increase or decrease in proteins identified in both groups. A total of 3,948 proteins were identified, of which 2,258 proteins were significantly differentially expressed between the two groups, with 1,151 proteins with increased and 1,107 proteins with decreased expression (Figure 2A). Among them, proteins related to NSCs whose expression was significantly decreased in differentiation included Ki-67, NES, SOX2, and VIM, and proteins significantly increased in differentiation were GFAP, MANF, and mature neuronal marker MAP2, as well as aldehyde dehydrogenase, which metabolizes DA aldehyde metabolites in the brain thus preventing neurodegeneration and is involved in DAn differentiation and survival (Villa et al., 2009; Chiu et al., 2015; Grünblatt and Riederer, 2016). Furthermore, although TH was not identified in all differentiation samples, two enzymes involved in the synthesis of the TH cofactor tetrahydrobiopterin which is required for DA production, dihydropteridine reductase and sepiapterin reductase (Meiser et al., 2013), were increased in the differentiated cell group. Early neuronal marker TUBB3 was non-significantly increased (*q* > 0.05). The protein encoded by all other genes identified in the NGS analysis were either not found or not significantly differentially expressed in the proteomic study. For functional analysis, the *q* value was set to < 0.01 in order to increase rigor. Within this range, 1,012 genes were differentially expressed, 496 of them with increased expression and 516 genes with decreased expression (Figure 2B).

The proteins with the biggest fold change in differentiated cells were neuromodulin (GAP43), a protein involved in neuronal and axonal growth and regeneration which is upregulated by BDNF and whose expression has been found to be decreased in PD patient brains (Saal et al., 2017; Chung et al., 2020), doublecortin-like kinase 1 (DCLK1), a protein kinase part of the doublecortin family that participates in neuronal migration and neurogenesis (Shu et al., 2006; Nawabi et al., 2015; Patel et al., 2016), and eukaryotic elongation factor 1 alpha 2 (EEF1A2), a protein implicated in protein translation elongation and autophagy that inhibits apoptotic cell death and may be important for DAn survival (Khwanraj et al., 2016; Prommahom and Dharmasaroja, 2021). These three proteins, along with vesicle amine transport 1 like (VAT1L), were also the only proteins among those with the largest fold change in differentiated cells whose genes also exhibited a significant fold change in the NGS study (data not shown). The proteins with the biggest fold change increase in dividing cells were sentrin-specific protease 3 (SENP3), lysine-specific demethylase 3B (KDM3B), and 7SK snRNA methylphosphate capping enzyme (MEPCE) (Figure 2C). All genes encoding the top 10 proteins with the largest fold change were identified in the NGS with significant fold changes and higher expression in dividing hVM1 clone 32 cells, except for ALB, which was more highly expressed in differentiated hNSCs (data not shown).

The top canonical pathways included fatty acid beta-oxidation I, valine degradation I, ethanol degradation IV, glutaryl-coenzyme A (CoA) degradation, tryptophan degradation III, and ketogenesis, all pathways involving energy metabolism and occurring mostly or entirely in the mitochondria and peroxisomes (MetaCyc, 2021). Mitochondria and peroxisomes are known to be dysfunctional in PD (Exner et al., 2012; Jo et al., 2020), thus emphasizing the importance of their functionality in DAn health. Moreover, fatty acids are neuroprotective (Parga et al., 2018), and aldehyde dehydrogenase, which is involved in DA metabolism and associated with DAn generation, is a key enzyme in the ethanol degradation IV pathway (Villa et al., 2009; Grünblatt and Riederer, 2016; MetaCyc, 2021). Ethanol is known to have an effect on DAn (Melis et al., 2009; Ma and Zhu, 2014). As well, the nuclear factor erythroid 2-related factor 2 (NFE2L2/NRF2)-mediated oxidative stress response and BAG family molecular chaperone regulator 2 (BAG2) signaling pathways have both been implicated in PD (Jazwa et al., 2011; Che et al., 2013; Qin et al., 2016; Todorovic et al., 2016) (Figure 2D).

One of the top networks of the functional analysis involved five of the proteins with the biggest fold change, namely branched-chain alpha-keto acid dehydrogenase E1 component beta (BCKDHB), EEF1A2, MEPCE, prothymosin alpha (PTMA), and SENP3, and the diseases and disorders of cancer, organismal injury and abnormalities, and gastrointestinal disease (Figure 2E). The top diseases and disorders were cancer, endocrine system disorders, organismal injury and abnormalities, gastrointestinal disease, and neurological disease, the last three of which coincide with PD itself and the non-motor symptoms of the disorder affecting the gut (Chaudhuri and Schapira, 2009; Kalia and Lang, 2015) (Figure 2F, top). Cancer and diabetes mellitus, one of the most common endocrine system disorders, have been shown to have pathogenic overlaps with PD, thus the involvement of the same proteins in PD and these two prevalent diseases (Khwanraj et al., 2016; Sergi et al., 2019). Notably, the proteins which had the biggest fold change in differentiated hVM1 clone 32 cells, GAP43, DCLK1, and EEF1A2, are implicated in several cancers (Patel et al., 2016; Giudici et al., 2019; Zheng et al., 2019).

Among the top 20 biological functions were cellular compromise, energy production, cell death and survival, nervous system development and function, cell morphology, and cellular function and maintenance, all important functions in the maintenance and survival of DAn (Figure 2F, bottom).

The top upstream regulators identified in proliferating and differentiated cells were involved in all of the aforementioned diseases, canonical pathways, and functions, such as cancer, diabetes mellitus, energy metabolism, glucose metabolism, lipid metabolism, and cell survival. In proliferating cells specifically, top upstream regulators also included oncogenes, hormones, as well as insulin and proteins associated with insulin regulation. Of particular interest were Myc proto-oncogene (MYC), involved in the cell cycle and apoptosis (Fults et al., 2002; Hoffman and Liebermann, 2008), as the hVM1 clone 32 cells are immortalized with MYC. Furthermore, mitogen-activated protein kinase kinase kinase kinase 4 (MAP4K4), one of the members of the MAP kinase kinase kinase kinase family, which is involved in the immune response and inflammation (Chuang et al., 2016), and mammalian target of rapamycin (MTOR), which was inhibited, controls cell growth, protein synthesis, and survival (Lan et al., 2017). In differentiated cells specifically, top upstream regulators included tumor suppressors, as well as proteins involved in calcium signaling, cell growth and division, neurite growth, and apoptosis. Several demethylases were also among the top upstream regulators indicating epigenetic regulation. Of particular interest were GDNF, a NTF involved in DAn survival and PD pathology (Allen et al., 2013), platelet-derived growth factor subunit BB (PDGF BB), a PDGF subunit which is implicated in neurogenesis (Mohapel et al., 2005; Yang et al., 2013), as well as amyloid-beta precursor protein (APP), a protein important for neuronal growth and migration, neurogenesis, and synaptogenesis (Coronel et al., 2019). Another top upstream regulator in differentiated cells was NFE2L2/NRF2. This protein, which was activated, has been described as a key protein in the antioxidant response and plays an important role in PD as well as other neurodegenerative diseases (Todorovic et al., 2016). The phosphoinositide 3-kinase (PI3K) family include kinases that are essential to the PI3K/Akt pathway, which has been shown to regulate autophagy, neuronal survival, proliferation, and differentiation, neurogenesis, and synaptic plasticity (Long et al., 2021). Moreover, several peroxisome proliferator-activated receptor (PPAR) proteins, namely PPAR A, D, and G (PPARA, PPARD, and PPARG), were among the top upstream regulators, and PPARA and PPARG were activated. The PPARs are receptors involved in many biological functions such as cell differentiation, DA signaling, energy metabolism, glucose metabolism, lipid metabolism, mitochondrial function, neuroinflammation, and oxidative stress. By providing neuroprotection through these various functions, PPAR agonists are a potential therapeutic target in PD (Chaturvedi and Beal, 2008; Wójtowicz et al., 2020) (Figure 2G).

The proteomic study broadened the scope of factors involved in DAn differentiation and survival as well as PD. Therefore, we decided to reanalyze the NGS data looking for genes involved in the aforementioned functions and pathways, including cancer, glucose, energy, and lipid metabolism, calcium signaling, and other genes of interest, focusing only on genes with a positive fold change in differentiated cells. A list of these genes can be found in Supplementary Table S3.

### Visualization of hVM1 Clone 32 Cell Markers *In Vitro* and *In Vivo*

**FIGURE 3:**
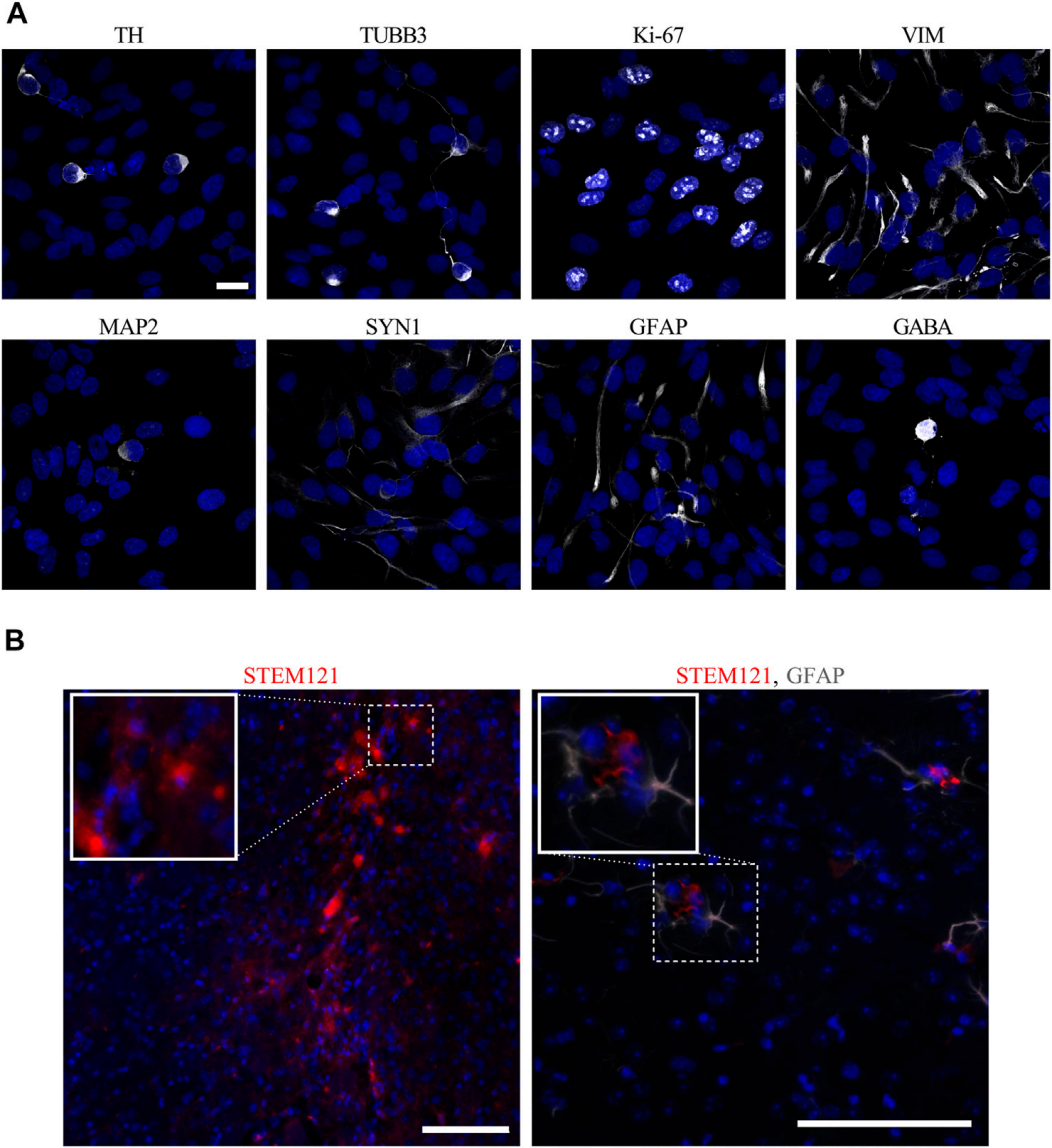
*In vitro* and *in vivo* images of hVM1 clone 32 cell markers. **(A)** After 7 days of differentiation *in vitro*, hVM1 clone 32 cells express a range of proteins including TH, TUBB3, Ki-67, VIM, MAP2, SYN1, GFAP, and GABA (all in white). Nuclei were stained with DAPI (blue). Scale bar = 20 μm. **(B)** Surviving transplanted hNSCs in the Str 4 months post-transplant as marked by STEM121 in red which stains human-specific cytoplasm **(left)**, and astrocytes as marked by GFAP in grey near the transplanted cells **(right)**. Nuclei were stained with DAPI (blue). Scale bars = 100 μm.

In order to look at morphology and verify major NSC and DAn protein markers, immunocytochemistry was performed on hVM1 clone 32 cells after 7 days of differentiation *in vitro*. In concordance with Ramos-Moreno et al. (2012b), which originally described the hVM1 clone 32 cells, the cells expressed DAn marker TH and immature neuronal marker TUBB3. At this stage of differentiation, the hNSCs still expressed NSC markers Ki-67 and VIM, and began to express mature neuronal markers like MAP2 and SYN1. Although the differentiated hVM1 clone 32 cells tend to generate DAn because of their tissue of origin, the ventral mesencephalon, in addition to their purposeful direction of differentiation toward DAn based on the factors in the differentiation media, namely GDNF and dibutyryl cAMP, the culture was not entirely homogeneous; markers for astrocytes (GFAP) and GABAergic neurons (GABA), were found in the culture after 7 days of differentiation, reflecting NGS and proteomic data (Figure 3A).

The hVM1 clone 32 cells were subsequently transplanted in the Str of middle-aged Parkinsonian mice. Four months post-transplant, there was little to no surviving hVM1 clone 32 cells found in the Str of transplanted mice. No hNSCs were found in other CNS regions such as the SNpc or Hip either (Figure 3B).

### Striatal and Nigral Tyrosine Hydroxylase Expression was Decreased in all Parkinsonian Mice

**FIGURE 4:**
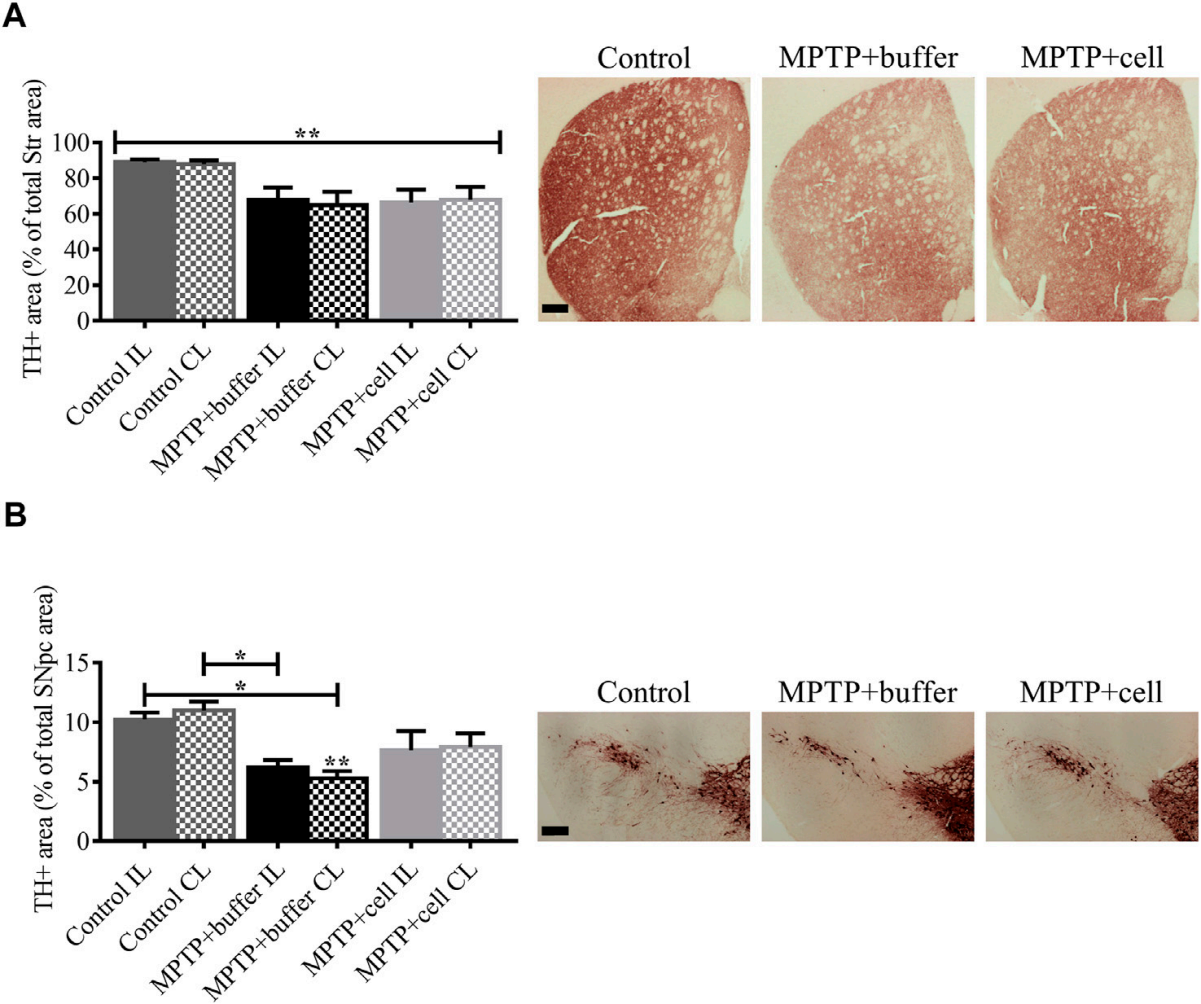
Diminution of striatal and nigral TH expression in Parkinsonian mice. **(A)** In the Str, all MPTP-lesioned mice tended to have around 25% less TH+ fiber density compared to controls. Control *n* = 5, MPTP + buffer *n* = 4, MPTP + cell *n* = 5. Kruskal-Wallis test followed by Dunn´s post-hoc test. **(B)** Nigral TH+ area decreased by 46% in buffer-treated mice (*p* < 0.05) and by 27% in hNSC-transplanted mice, compared to control animals. Control *n* = 5, MPTP + buffer *n* = 4, MPTP + cell *n* = 5. One-way ANOVA followed by Tukey´s post-hoc test. **(A,B)**: * = *p* < 0.05, ** = *p* < 0.01. * = compared to same brain hemisphere of control. Data are expressed as mean ± standard error of the mean. Scale bars = 200 μm.

Four months post-transplant, mouse brains were analyzed for TH immunoreactivity in the Str and SNpc, the two main regions affected in PD. In the Str, the means of the groups in terms of TH expression were statistically different (*p* < 0.01). Although not statistically significant, all MPTP-lesioned mice had approximately 25% less TH+ fiber density in the Str compared to control animals (Figure 4A). In the SNpc, there was a 46% decrease in TH+ area in buffer-treated mice compared to controls (*p* < 0.05). By contrast, hNSC-treated animals tended to have a 27% decrease in nigral TH+ area compared to control mice and a 26% increase in TH expression in the SNpc compared to buffer-transplanted mice (Figure 4B). Expression of TH was markedly decreased in both the Str and SNpc and this diminution of TH+ immunostaining was not alleviated by hVM1 clone 32 cell transplant.

### Activity and Gait Changes Were Improved in hVM1 Clone 32-Transplanted Mice

**FIGURE 5:**
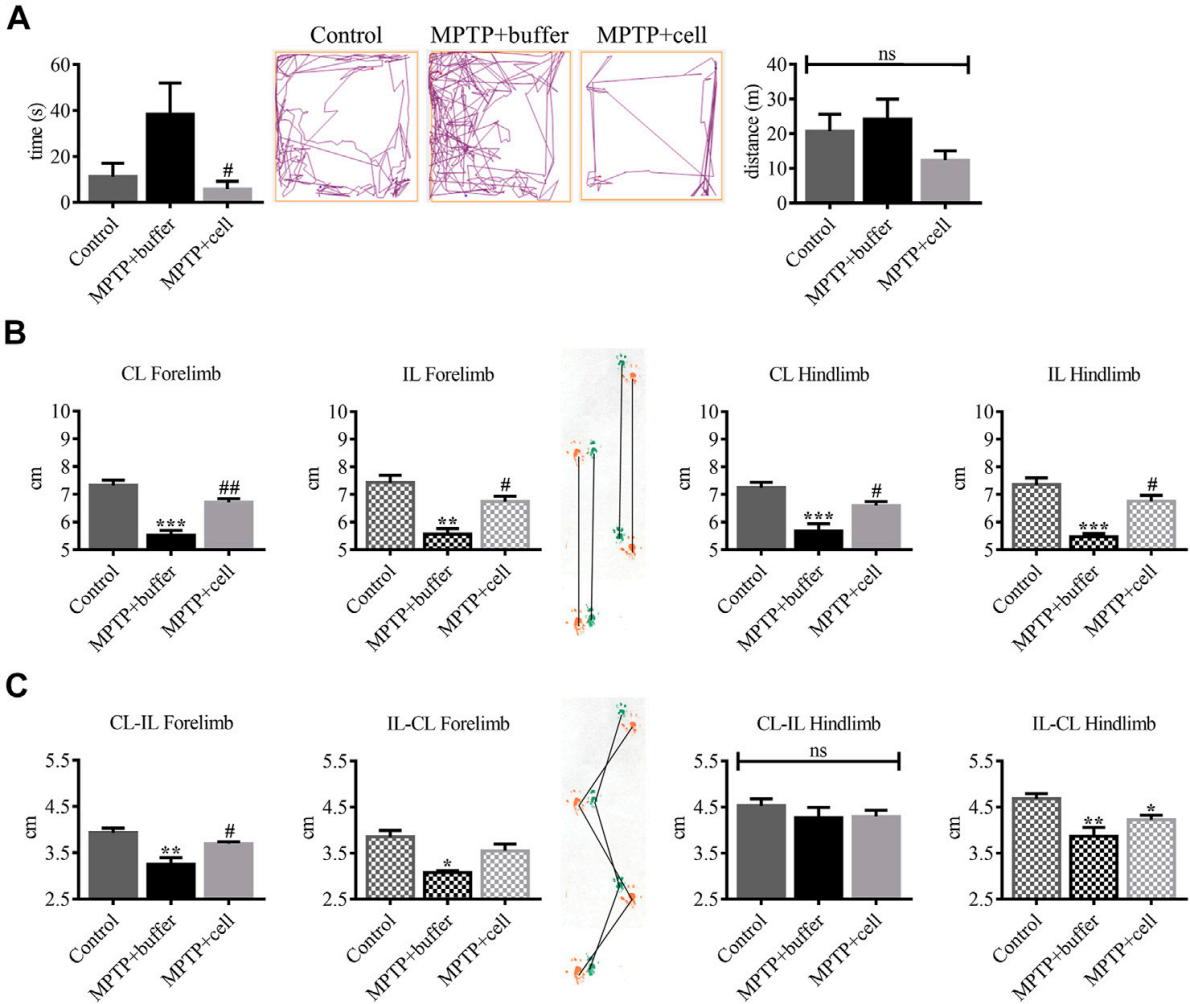
Behavioral improvement was observed in hNSC-transplanted mice. **(A, left)** Buffer-treated animals had a tendency to spend 71% more time in the center of the box compared to controls, while NSC-transplanted mice spent 85% less time in the center compared to those that received buffer (*p* < 0.05). Control *n* = 5, MPTP + buffer *n* = 4, MPTP + cell *n* = 5. One-way ANOVA followed by Tukey´s post-hoc test. **(A, right)** All experimental groups had the same distance traveled. Control *n* = 5, MPTP + buffer *n* = 4, MPTP + cell *n* = 5. Kruskal-Wallis test. **(B)** Forelimb and hindlimb stride lengths decreased by approximately 24% in buffer-treated animals compared to controls (*p* < 0.01), and hNSC transplant led to a 17% increase in all stride lengths measured compared to the vehicle group (*p* < 0.05). **(C)** Forelimb stride width was around 19% shorter in buffer-treated mice compared to control animals (*p* < 0.05) and NSC transplant tended to increase forelimb stride width by 13% compared to mice that received buffer, although only attaining significance (*p* < 0.05) on one side measured. CL-IL hindlimb stride width was unchanged among the three experimental groups and when compared to the control group, IL-CL hindlimb stride width was reduced in all MPTP-lesioned mice, although to a greater extent in buffer-treated (17%; *p* < 0.01) than cell-transplanted animals (10%; *p* < 0.05). **(B,C)**: Control *n* = 7, MPTP + buffer *n* = 3, MPTP + cell *n* = 5. One-way ANOVA followed by Tukey’s post-hoc test. **(A–C)**: *, ^#^ = *p* < 0.05, **, ^##^ = *p* < 0.01, *** = *p* < 0.001, ns, not significant. * = compared to control, ^#^ = compared to MPTP + buffer. Data are expressed as mean ± standard error of the mean.

Several mouse behaviors were measured to examine the effects of hVM1 clone 32 transplant 4 months post-transplant. In the OFT, there were no significant differences among the three experimental groups in terms of time spent grooming, time spent rearing, urination, and defecation (data not shown). As for time spent in the center of the OFT box, buffer-treated mice tended to spend around 71% more time in the center compared to controls, indicating hyperactivity. This increased time spent in the center was decreased by 85% in hNSC-transplanted animals (*p* < 0.05). There were no differences among the three experimental groups in terms of distance traveled, suggesting a lack of bradykinesia in the MPTP-lesioned mice (Figure 5A). The PPT was performed to analyze changes in gait between the three groups of animals. All stride lengths were decreased by an average of 24% in buffer-treated mice compared to controls (*p* < 0.01) and were then increased by 17% in cell-transplanted mice compared to those that received buffer (*p* < 0.05) (Figure 5B). Forelimb stride width was reduced by approximately 19% in buffer-treated animals compared to controls (*p* < 0.05), while with hNSC transplant it tended to increase by 13% compared to those that received buffer, but only reached statistical significance (*p* < 0.05) on one side. By contrast, CL-IL hindlimb stride width was similar in all three experimental groups while IL-CL hindlimb stride width was decreased in buffer-treated mice by 17% (*p* < 0.01) and in cell-transplanted animals by 10% (*p* < 0.05), compared to controls (Figure 5C).

### Dopamine Functionality, Neuroinflammation, and Motor Cortex Changes Influenced Transplant Outcome

**FIGURE 6:**
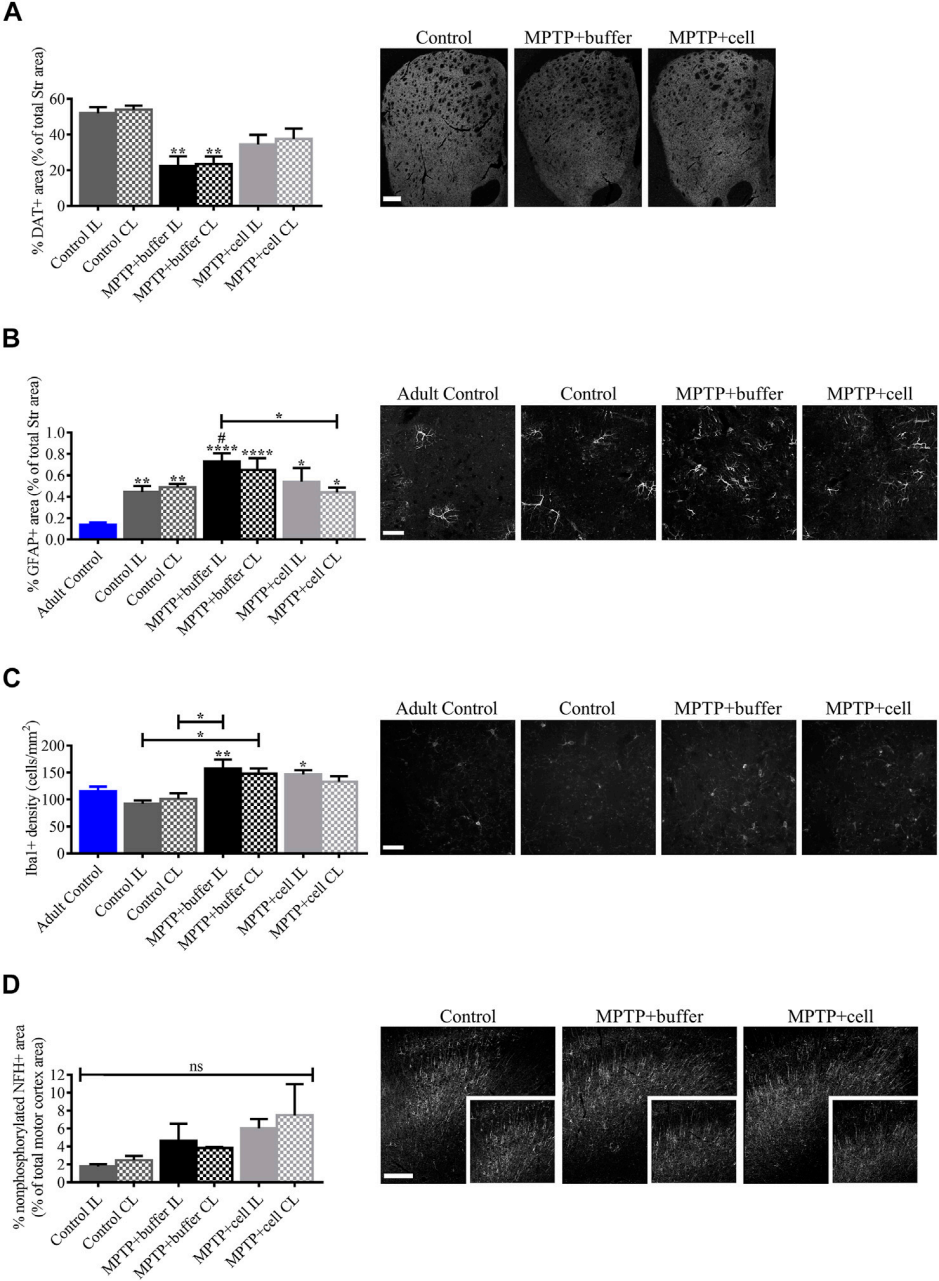
Evaluation of factors that potentially influenced hNSC transplant including DA transport, inflammation, and motor cortex alterations. **(A)** Compared to controls, DAT immunostaining decreased by approximately 57% in buffer-treated mice (*p* < 0.01) and non-significantly by around 32% in cell-transplanted animals, although DAT+ area tended to be increased by 36% in hNSC-treated mice compared to those that received buffer. Control *n* = 3, MPTP + buffer *n* = 4, MPTP + cell *n* = 4. **(B)** Striatal expression of GFAP tended to be higher in buffer-treated animals, reaching statistical significance on the IL side (*p* < 0.05), and was decreased on the CL side of hNSC-transplanted mice compared to the IL side of buffer-treated animals (*p* < 0.05). When compared to adult control mice, all middle-aged mice had increased GFAP expression in the Str (*p* < 0.05). Adult Control *n* = 3, Control n = 5, MPTP + buffer *n* = 4, MPTP + cell *n* = 3. One-way ANOVA followed by Tukey’s post-hoc test. *, ^#^ = *p* < 0.05, ** = *p* < 0.01, **** = *p* < 0.0001. * = compared to adult control, ^#^ = compared to same brain hemisphere of middle-aged control. Data are expressed as mean ± standard error of the mean. Scale bar = 50 μm. **(C)** Iba1+ microglial populations were increased in the Str of all Parkinsonian mice (*p* < 0.05), and age did not affect microglial density. Adult Control *n* = 5, Control *n* = 4, MPTP + buffer *n* = 5, MPTP + cell *n* = 5. One-way ANOVA followed by Tukey´s post-hoc test. * = *p* < 0.05, ** = *p* < 0.01. * = compared to same brain hemisphere of middle-aged control. Data are expressed as mean ± standard error of the mean. Scale bar = 50 μm. **(D)** In the motor cortex, nonphosphorylated NFH+ area tended to be increased in animals intoxicated with MPTP by 50–69% compared to control mice. Control *n* = 3, MPTP+buffer *n* = 3, MPTP + cell *n* = 3. **(A,D)**: One-way ANOVA followed by Tukey’s post-hoc test. ** = *p* < 0.01, ns, not significant. * = compared to same brain hemisphere of control. Data are expressed as mean ± standard error of the mean. Scale bars = 200 μm.

Because of the surprising discrepancy between TH expression in the Str and behavioral symptoms upon administration of MPTP and hVM1 clone 32 transplant, it was important to explain this occurrence by analyzing several potential contributors to this effect. The functionality of the striatal TH+ fibers, neuroinflammation, and motor cortex changes, were explored. First, another marker for dopaminergic terminals, namely DAT, was tested. Striatal expression of DAT was significantly decreased in buffer-treated animals by around 57% compared to controls (*p* < 0.01). Moreover, DAT+ area tended to be reduced by approximately 32% in the Str of cell-transplanted mice compared to control animals; however, DAT expression tended to be 36% greater in hNSC-treated animals compared to buffer-treated mice (Figure 6A).

Second, neuroinflammation, and more specifically GFAP and Iba1 expression in the Str, was examined in order to see a potential role for astrocytes and activated microglia. Astroglial immunoreactivity in the Str tended to increase in buffer-treated mice compared to controls, although this was only statistically significant on the IL side (*p* < 0.05). Microglial populations were increased in animals that received buffer and on the IL side of hNSC-transplanted mice compared to control animals (*p* < 0.05). Furthermore, striatal GFAP expression decreased on the CL side of cell-transplanted mice compared to the IL side of those treated with buffer (*p* < 0.05). Interestingly, when comparing animals from each of the three experimental groups, which were approximately 16 months old at experiment endpoint, to adult (nine month-old) control mice submitted to the same protocol, there was a significant increase in striatal GFAP (*p* < 0.05), and not Iba1, expression (Figures 6B,C).

Lastly, nonphosphorylated neurofilament populations were analyzed in the motor cortex, which is connected to the Str, in order to study axonal transport and function. Although not statistically significant, there tended to be higher expression of nonphosphorylated NFH in the motor cortex of MPTP-treated mice compared to controls, with an increase of 50% in buffer-treated and 69% in hNSC-transplanted animals (Figure 6D).

Therefore, further characterization of hVM1 clone 32 cells through gene and protein expression analyses revealed that these are true hNSCs that have the capability of generating DAn. These *in vitro* findings also supported the notion that multiple factors affect PD and DAn, with this study emphasizing the role of mitochondrial and peroxisome function, as well as glucose and lipid metabolism. Although hVM1 clone 32 cells did not survive when transplanted in middle-aged mice, most behavioral symptoms were alleviated by hNSC transplant, which was reinforced by a tendency for striatal DAT expression to be higher in cell-treated animals compared to those that received buffer. Additionally, nigrostriatal TH expression was notably decreased and cortical nonphosphorylated NFH expression was increased in all Parkinsonian mice.

## Discussion

Our data show that hVM1 clone 32 cells are hNSCs that have DAn features upon differentiation *in vitro*, and when these cells are transplanted in middle-aged Parkinsonian mice, there is an improvement in both motor and non-motor functioning, which is supported by a tendency of restored functional dopaminergic striatal terminals. Moreover, MPTP administration in these mice leads to notable, although minimal, striatal TH degeneration and motor cortical axonal transport alterations, and CRT was not able to be fully effective in middle-aged MPTP-intoxicated mice because of increased neuroinflammation.

Both NGS and proteomic analyses of differentiated hNSCs allowed us to deviate from classic, DA-centered markers and pathways involved in DAn survival and PD pathology, and emphasize the importance of less-studied factors influencing DAn such as calcium signaling, mitochondrial and peroxisome function, glucose and lipid metabolism, and oxidative stress, thus confirming that PD is multifactorial.

With all of the evidence presented that hVM1 clone 32 cells can generate DAn, it is important to note that 7 days of differentiation is still an early stage of differentiation so it is not out of the ordinary that NSC markers were still expressed. The differentiated hNSC culture was also heterogeneous, with the hVM1 clone 32 cells expressing both genes and proteins associated with other CNS populations such as astrocytes, oligodendrocytes, serotonergic neurons, GABAergic neurons, glutamatergic neurons, and cholinergic neurons. In the future, it would be interesting to see the effects of a longer hNSC differentiation.

The amount of surviving transplanted cells in the brain of hNSC-transplanted mice was minimal, yet behavioral improvement was observed, which is in line with other stem cell therapy studies (Fu et al., 2015). Cell survival is a constant problem in CRT, with approximately 95% of transplanted cells dying shortly after grafting in experimental PD animals and PD patients (Emgard et al., 2003; Rafuse et al., 2005; Stoker and Barker, 2016). The CSA protocol was not the cause of transplanted cell death in this study as the equivalent protocol was used in Parkinsonian rats where the hVM1 clone 32 cells survived 2 months post-transplant (Ramos-Moreno et al., 2012b). Furthermore, cell survival has been demonstrated to be negligible in CNS transplants in mice when compared to rats (Roberton et al., 2013). Although the hVM1 clone 32 cells did not survive transplantation in this study, we were still able to explore their effects in middle-aged Parkinsonian mice.

The diminution of TH expression in the Str observed, although notable, was surprising as most studies find that middle-aged and aged Parkinsonian mice exhibit a more substantial decrease in TH expression in the Str and SNpc as well as striatal DA content, and demonstrate more severe behavioral deficits (Date et al., 1990; Ohashi et al., 2006; Guan et al., 2016). However, most of these studies used a larger dose of MPTP. As well, none of the studies used the exact same acute MPTP protocol in C57BL/6 mice of the same vendor, and it has been shown that different MPTP protocols lead to varied nigrostriatal damage, and that mice of the same strain from different providers show diverse susceptibility to MPTP (Jackson-Lewis and Przedborski, 2007). Genetics perhaps plays a role because older mice have higher lethality when given MPTP so the ones that do survive may have a stronger resistance to MPTP and therefore there is less damage to their nigrostriatal pathway.

Spontaneous dopaminergic sprouting has been shown to occur in the Str between 10 days and 5 months post-MPTP administration in mice between the ages of 8 and 10 weeks. However, dopaminergic recovery has not observed in the Str of older (eight month-old) mice, nor in the SNpc of both younger and older MPTP-treated mice (Ho and Blum, 1998; Mitsumoto et al., 1998; Bezard et al., 2000; Jakowec et al., 2004).

In the present study, behavioral deficits were observed in buffer-treated mice in terms of increased hyperactivity and changes in gait; these behavioral aspects tended to be improved by hNSC transplant. Furthermore, parameters measuring anxiety and locomotion were not affected by MPTP treatment or hNSC transplant. Depletion of DA in the prefrontal cortex has been shown to increase hyperactivity and decrease anxiety, and this hyperactivity is due to disinhibition and attention deficit (Rousselet et al., 2003). Attention deficit and impulsivity occur in some PD patients (Nieoullon, 2002; Kehagia et al., 2014; Nombela et al., 2014). Striatal TH fiber abundance did not mimic the behavioral deficits in buffer-treated animals, but the decrease of DAT expression in the Str did follow this trend. This reveals that immunostaining of TH to analyze dopaminergic fiber degeneration is not a functional assessment, and in this case, striatal expression of DAT, stride length, stride width, and time spent in the center, were able to detect differences between buffer-treated and control animals, all of which striatal TH expression studies were not able to do. Also, only behavioral studies, and not immunostaining, were able to uncover statistically significant differences between buffer- and cell-treated groups. Furthermore, in mouse models of PD, behavior does not always reflect disease pathology in the brain (Zhang et al., 2017; Hunn et al., 2019). In this study, improvement of behavioral deficits in cell-transplanted mice with no significant increase in either TH or DAT expression, compared to animals injected with buffer, could be due to a hyperdopaminergic state, where enhanced dopaminergic neurotransmission leads to behavioral improvement while PD pathology remains (Hunn et al., 2019).

Both TH and DAT are used to mark DAn; however, TH marks all catecholaminergic neurons, one of them being DAn, while DAT specifically marks DAn. Tyrosine hydroxylase is the rate-limiting enzyme of DA synthesis, while DAT reuptakes DA at the synapse, thus controlling the availability of DA. In addition, the two proteins differ in expression levels in the Str and SNpc (Miller et al., 1999; Seifert and Wiener, 2013; Vaughan and Foster, 2013; Salvatore et al., 2016). Although TH works in DA synthesis, it does not reveal the activity of DA, which DAT does, as changes in DAT expression levels indicate variations in the transport and release of DA, and can explain the function of these dopaminergic striatal terminals. In addition, it has been hypothesized that abnormal transport of DA can lead to the development of diseases such as PD (Vaughan and Foster, 2013). Both TH and DAT are decreased in the Str of PD patients. However, there is evidence of a compensatory mechanism as a response to striatal DA depletion in PD patients and animals involving augmented synthesis, release and turnover of DA and TH, and increased TH expression and decreased DAT expression in the Str (Miller et al., 1999; Blesa et al., 2017). Interestingly, the visualization of DAT in the Str using positron emission tomography and single photon emission computed tomography imaging such as DATscan is used to help in the diagnosis of PD, thus emphasizing the importance of DAT expression and function, rather than TH expression in PD pathology (Seifert and Wiener, 2013). Most PD and CRT studies quantify nigrostriatal population changes via TH immunostaining (Ang, 2006; Kriks et al., 2011; Stoker and Barker, 2016), but it is clear from this study that TH and DAT expression do not always follow the same pattern. The function of DAT has been associated with the regulation of locomotor activity, which would support the connection between behavioral impairment and expression of DAT, rather than TH (Chotibut et al., 2012). Ultimately, it is important to expand the tests done in experimental PD and CRT as TH, although a good indicator, may not always be the best marker for nigrostriatal degeneration and/or improvement.

Inflammation is known to play an essential role in PD (Sarkar et al., 2016). In this study, buffer-treated animals tended to have higher amounts of striatal astrocytes, while all middle-aged mice had significantly increased striatal inflammation compared to younger controls. This emphasizes just how important the age of the transplant recipient is as CRT is more successful in younger patients (Stoker and Barker, 2016; Yasuhara et al., 2017) perhaps in part due to the fact that higher inflammation levels hamper its effectiveness. Moreover, while microglia were more abundant in the Str of all Parkinsonian mice, the transplant had no effect on microglial activation.

To further support a functionality problem, nonphosphorylated NFH expression in the motor cortex was increased in all MPTP-treated animals compared to control mice. It has been described that PD patients show neuronal loss in the motor cortex (MacDonald and Halliday, 2002). As well, there are structural and functional changes in the motor cortex of PD patients; for example, monoamine deficiencies and both hyperactivity and hypoactivity have been observed (Blesa et al., 2017; Burciu and Vaillancourt, 2018). Furthermore, it has recently been proposed that PD comprises a top-down mechanism meaning that pathological changes in the cortex eventually lead to nigrostriatal neurodegeneration (Foffani and Obeso, 2018). Neurofilament heavy is one of the three subunits of neurofilaments that are present in neurons. Phosphorylation of NFH is key for axonal transport, axonal caliber, axonal diameter, axonal plasticity, and neuronal morphology, and accumulation of neurofilaments as well as variations in modifications to neurofilaments like phosphorylation are present in neurodegenerative diseases such as PD (Nixon and Sihag, 1991; Pant and Veeranna, 1995; Kashiwagi et al., 2003; Liu et al., 2011; Kirkcaldie and Dwyer, 2017). Therefore, an increase in nonphosphorylated NFH suggests a change in axonal function in all MPTP-treated mice in a region of the brain connected to the Str and responsible for movement, although with opposite effects on gait in buffer- and cell-treated animals.

For all immunostaining and behavioral data, the IL and CL sides were affected equally in all three experimental groups, with one exception. Hindlimb stride width was significantly shorter on the IL-CL side in MPTP-treated mice, while no significant changes were seen among the control and MPTP-administered mice on the CL-IL side.

Many experimental CRT for PD studies are done in adult animals, using rodents from 2 to 4 months old (Courtois et al., 2010; Ramos-Moreno et al., 2012a; Ramos-Moreno et al., 2012b; Zuo et al., 2015; Zuo et al., 2017). However, this translates poorly into clinical situations as PD is a pathology that affects the elderly and age-focused studies need to be more thoroughly explored. Hence, this experimental CRT study was done in middle-aged Parkinsonian mice using the hNSC line hVM1 clone 32. Although there is a lack of data on potential treatments in middle-aged or aged Parkinsonian animals, a few studies have demonstrated some success. The injection of GDNF in the SNpc of 18- and 24-month old Parkinsonian rats led to a significant recovery of TH+ cells in the SNpc, and striatal DA and 3,4-dihydroxyphenylacetic acid levels (Fox et al., 2001). As well, Ourednik et al. (2002), treated 20-month old mice with MPTP and transplanted them with murine NSCs above the SN/ventral tegmental area 1 week or 4 weeks later. There were no changes in behavior in any of the groups throughout the experiment, and 3 weeks post-transplant, TH expression was recovered in the Str and SNpc of hNSC-transplanted mice and expression of DAT was restored to almost control-like levels in these two brain regions (Ourednik et al., 2002).

Our results indicate that differentiated hVM1 clone 32 cells have the profile of DAn required for successful transplant. Although these hNSCs could never be used in clinical trials due to their fetal origin, *v*-*myc* immortalization, and need for recipient immunosuppression, their characterization helps in understanding and developing NSCs for clinical CRT to treat PD as well as exploring their capability as a source of NTFs in the form of conditioned media (Fričová et al., 2020). When transplanted in their undifferentiated state, although nigrostriatal pathway degeneration was not significantly improved, motor and non-motor impairments were prevented by hNSC transplant in middle-aged Parkinsonian mice, which when translating to human PD cases, is perhaps the most important. Further research is warranted, but these data confirm the efficacy of hNSC transplantation in the treatment of PD and stress the importance of performing experimental CRT studies in aged Parkinsonian mice.

As PD is principally described as a loss of DAn and DA, therapeutic strategies have mostly focused on the preservation or restoration of the nigrostriatal pathway. Our findings, both *in vitro* and *in vivo*, demonstrate that the clinical outcome of PD in humans is far more complex than just nigrostriatal degeneration and motor symptoms, and is indeed multifactorial and includes growth factors and NTFs, mitochondrial and peroxisome function, glucose and lipid metabolism, non-motor neurological aspects, DA functionality, neuroinflammation, and motor cortex changes, all of which could lead to new targets and routes for the treatment of PD.

## Data Availability

The NGS and proteomic datasets presented in this study can be found online at https://doi.org/10.21950/4IXBTX and https://doi.org/10.21950/06EW6H, respectively. The remaining raw data supporting the conclusions of this article will be made available by the authors upon reasonable request. Supplementary Material includes lists of categorized genes differentially expressed in proliferative and differentiated hNSCs.
