# Supplementary material for: Multifactoriality of Parkinson’s Disease as Explored Through Human Neural Stem Cells and Their Transplantation in Middle-Aged Parkinsonian Mice
Source: Front Pharmacol. 2022 Jan 19;12:773925. doi: 10.3389/fphar.2021.773925 (PMC8807563; doi:10.3389/fphar.2021.773925)
Supplement: Supplementary file 1 [file DataSheet1.docx]

Supplementary Material

# Supplementary Tables

**Supplementary Table S1.** List of genes differentially expressed by dividing (blue) and differentiated (red) hVM1 clone 32 cells. Genes are categorized by their association with NSCs, immature neurons, mature neurons, DAn, factors and their receptors, cytokines and their receptors, astrocytes, oligodendrocytes, as well as serotonergic, GABAergic, glutamatergic, and cholinergic neurons.

| **Category** | **Gene symbol** | **Gene ID** | **Fold change** | **q value** |
| --- | --- | --- | --- | --- |
| NSCs | *MKI67* | ENSG00000148773 | -3.31 | 4.73E-24 |
|  | *NOTCH1* | ENSG00000148400 | -2.89 | 1.23E-16 |
|  | *HES1* | ENSG00000114315 | -2.43 | 1.20E-09 |
|  | *NES* | ENSG00000132688 | -2.29 | 1.08E-10 |
|  | *NOG* | ENSG00000183691 | -2.03 | 0.000214 |
|  | *BLBP* | ENSG00000164434 | -1.82 | 1.13E-05 |
|  | *VIM* | ENSG00000026025 | -1.74 | 1.10E-06 |
|  | *NUMB* | ENSG00000133961 | -0.72 | 0.0322 |
| Immature neurons | *STMN1* | ENSG00000117632 | -2.35 | 2.77E-10 |
|  | *DCX* | ENSG00000077279 | 1.07 | 0.00581 |
| Mature neurons | *SYN2* | ENSG00000157152 | 0.81 | 0.0192 |
|  | *CALB2* | ENSG00000172137 | 1.00 | 0.0448 |
|  | *NEUN* | ENSG00000167281 | 1.17 | 0.00124 |
|  | *SYN1* | ENSG00000008056 | 1.29 | 0.0158 |
|  | *NCAM2* | ENSG00000154654 | 1.34 | 0.000786 |
| DAn | *EN1* | ENSG00000163064 | -2.51 | 1.22E-14 |
|  | *EN2* | ENSG00000164778 | -2.38 | 6.39E-08 |
|  | *OTX2* | ENSG00000165588 | -1.57 | 6.05E-06 |
|  | *DRD2* | ENSG00000149295 | 1.00 | 0.0102 |
|  | *WNT3A* | ENSG00000154342 | 1.04 | 0.0180 |
|  | *WNT2* | ENSG00000105989 | 1.06 | 0.0297 |
|  | *DAT* | ENSG00000142319 | 1.18 | 0.0223 |
|  | *GIRK2* | ENSG00000157542 | 1.19 | 0.00152 |
|  | *ALDH1A2* | ENSG00000128918 | 1.20 | 0.000892 |
|  | *ALDH1A1* | ENSG00000165092 | 1.20 | 0.00281 |
|  | *PAX5* | ENSG00000196092 | 1.25 | 0.00111 |
|  | *SLC6A2* | ENSG00000103546 | 1.31 | 0.00310 |
|  | *DRD3* | ENSG00000151577 | 1.32 | 0.000901 |
|  | *MYT1L* | ENSG00000186487 | 1.33 | 0.000204 |
|  | *LMX1B* | ENSG00000136944 | 1.34 | 0.00103 |
|  | *BMP5* | ENSG00000112175 | 1.36 | 0.00182 |
|  | *LMX1A* | ENSG00000162761 | 1.44 | 0.000189 |
|  | *PITX2* | ENSG00000164093 | 1.45 | 0.0105 |
|  | *BMP2* | ENSG00000125845 | 1.76 | 0.00622 |
| **Category** | **Gene symbol** | **Gene ID** | **Fold change** | **q value** |
|  | *PITX3* | ENSG00000107859 | 1.78 | 0.0121 |
|  | *TH* | ENSG00000180176 | 2.55 | 2.41E-07 |
|  | *DLK1* | ENSG00000185559 | 3.52 | 1.87E-16 |
| Factors and their receptors | *VEGFR2* | ENSG00000128052 | -2.26 | 2.98E-11 |
|  | *MANF* | ENSG00000145050 | -2.25 | 1.02E-09 |
|  | *CNTF* | ENSG00000242689 | -1.90 | 0.0138 |
|  | *VEGFB* | ENSG00000173511 | -1.90 | 7.80E-08 |
|  | *NRP2* | ENSG00000118257 | -1.90 | 5.57E-09 |
|  | *VEGFA* | ENSG00000112715 | -1.42 | 1.17E-05 |
|  | *NRP1* | ENSG00000150630 | -0.83 | 0.0158 |
|  | *NTRK3* | ENSG00000140538 | 0.82 | 0.0150 |
|  | *NTRK1* | ENSG00000198400 | 0.96 | 0.0345 |
|  | *VEGFC* | ENSG00000150630 | 1.33 | 0.00194 |
|  | *GFRA2* | ENSG00000168546 | 1.34 | 0.00248 |
|  | *VEGFR1* | ENSG00000102755 | 1.38 | 0.000631 |
|  | *GFRA1* | ENSG00000151892 | 1.53 | 8.19E-05 |
|  | *NTRK2* | ENSG00000148053 | 1.55 | 5.42E-06 |
| Cytokines and their receptors | *MCP1* | ENSG00000108691 | -1.70 | 5.48E-05 |
|  | *SCF* | ENSG00000049130 | -1.25 | 0.000233 |
|  | *KIT* | ENSG00000157404 | 1.49 | 0.000148 |
|  | *CCR2* | ENSG00000121807 | 1.82 | 0.0151 |
|  | *PTGS2* | ENSG00000073756 | 2.33 | 0.0260 |
|  | *IL1B* | ENSG00000125538 | 2.53 | 0.00480 |
|  | *IL6* | ENSG00000136244 | 2.92 | 0.0382 |
| Astrocytes | *HES1* | ENSG00000114315 | -2.43 | 1.20E-09 |
|  | *S100B* | ENSG00000160307 | -1.97 | 3.95E-07 |
|  | *SLC1A3* | ENSG00000079215 | -1.65 | 5.29E-06 |
|  | *ALDH1L1* | ENSG00000144908 | 0.86 | 0.0326 |
|  | *GFAP* | ENSG00000131095 | 2.65 | 3.89E-09 |
| Oligodendrocytes | *MOBP* | ENSG00000168314 | 1.15 | 0.0118 |
|  | *MBP* | ENSG00000197971 | 1.20 | 0.00505 |
|  | *OPALIN* | ENSG00000197430 | 2.18 | 0.00116 |
| Serotonergic neurons | *SLC6A4* | ENSG00000108576 | 1.07 | 0.0186 |
|  | *TPH2* | ENSG00000139287 | 1.27 | 0.00269 |
| GABAergic neurons | *SLC6A1* | ENSG00000157103 | 1.07 | 0.0203 |
|  | *GABBR1* | ENSG00000204681 | 1.09 | 0.00480 |
|  | *GABBR2* | ENSG00000136928 | 1.32 | 0.000261 |
| Glutamatergic neurons | *SLC17A6* | ENSG00000091664 | 0.92 | 0.0405 |
|  | *GRIN2B* | ENSG00000273079 | 1.43 | 0.000233 |
| Cholinergic neurons | *CHAT* | ENSG00000070748 | 1.11 | 0.00847 |

**Supplementary Table S2.** Extended list of genes differentially expressed by dividing (blue) and differentiated (red) hVM1 clone 32 cells. Genes are categorized by their association with NSCs, proliferation and cell cycle, neurons, neural plasticity, NTFs and their receptors, astrocytes, oligodendrocytes, serotonergic, GABAergic, glutamatergic, and cholinergic neurons, as well as familial PD.

| **Category** | **Gene symbol** | **Gene ID** | **Fold change** | **q value** |
| --- | --- | --- | --- | --- |
| NSCs | *SOX21* | ENSG00000125285 | -3.61 | 4.59E-28 |
|  | *SOX1* | ENSG00000182968 | -3.55 | 4.29E-18 |
|  | *SFRP2* | ENSG00000145423 | -3.44 | 3.37E-27 |
|  | *FZD9* | ENSG00000188763 | -3.16 | 1.71E-08 |
|  | *HES5* | ENSG00000197921 | -2.90 | 0.0442 |
|  | *METRN* | ENSG00000103260 | -2.71 | 1.61E-16 |
|  | *BMI1* | ENSG00000168283 | -2.50 | 1.08E-10 |
|  | *GNL3* | ENSG00000163938 | -2.39 | 4.76E-09 |
|  | *TRAF4* | ENSG00000076604 | -2.33 | 5.01E-13 |
|  | *SLC2A1* | ENSG00000117394 | -2.26 | 2.55E-13 |
|  | *NR2F1* | ENSG00000175745 | -2.02 | 4.56E-09 |
|  | *SMARCA4* | ENSG00000127616 | -1.97 | 6.43E-11 |
|  | *CTNNB1* | ENSG00000168036 | -1.94 | 2.54E-08 |
|  | *ACVR2B* | ENSG00000114739 | -1.93 | 2.28E-08 |
|  | *SLC1A3* | ENSG00000079215 | -1.65 | 5.29E-06 |
|  | *NEPRO* | ENSG00000163608 | -1.60 | 0.000530 |
|  | *CDH2* | ENSG00000170558 | -1.59 | 3.96E-06 |
|  | *GATA2* | ENSG00000179348 | -1.58 | 0.00198 |
|  | *NFE2L2* | ENSG00000116044 | -1.55 | 6.33E-06 |
|  | *ZIC1* | ENSG00000152977 | -1.50 | 4.02E-05 |
|  | *NOTCH2* | ENSG00000134250 | -1.43 | 6.85E-06 |
|  | *MSI1* | ENSG00000135097 | -1.40 | 2.92E-05 |
|  | *SOX11* | ENSG00000176887 | -1.28 | 8.31E-05 |
|  | *MTURN* | ENSG00000180354 | -1.23 | 0.000305 |
|  | *MSI2* | ENSG00000153944 | -1.19 | 0.000343 |
|  | *RXRA* | ENSG00000186350 | -1.19 | 0.000331 |
|  | *ACVR1B* | ENSG00000135503 | -0.97 | 0.00380 |
|  | *TNC* | ENSG00000041982 | -0.96 | 0.00423 |
|  | *SLAIN1* | ENSG00000139737 | -0.90 | 0.0134 |
|  | *NPDC1* | ENSG00000107281 | -0.87 | 0.0318 |
|  | *ABCG2* | ENSG00000118777 | 1.04 | 0.00590 |
|  | *INHBA* | ENSG00000122641 | 1.09 | 0.0369 |
|  | *ROR2* | ENSG00000169071 | 1.11 | 0.00118 |
|  | *CDH1* | ENSG00000039068 | 1.19 | 0.00137 |
|  | *OCLN* | ENSG00000197822 | 1.27 | 0.00426 |
|  | *ACVR1C* | ENSG00000123612 | 1.27 | 0.00280 |
|  | *RUNX1* | ENSG00000159216 | 1.28 | 0.000334 |
|  | *PAX3* | ENSG00000135903 | 1.34 | 0.00209 |
|  | *PAX6* | ENSG00000007372 | 1.61 | 0.00313 |
|  | | | | |
| Proliferation and cell cycle | *SFRP2* | ENSG00000145423 | -3.44 | 3.37E-27 |
|  | *ZWINT* | ENSG00000122952 | -3.24 | 1.36E-17 |
|  | *PCNA* | ENSG00000132646 | -3.18 | 4.02E-17 |
|  | *GMNN* | ENSG00000112312 | -3.07 | 3.34E-15 |
|  | *CENPE* | ENSG00000138778 | -3.04 | 5.73E-15 |
|  | *PLK4* | ENSG00000142731 | -2.98 | 1.05E-15 |
|  | *PLK1* | ENSG00000166851 | -2.97 | 4.64E-21 |
|  | *CDC25A* | ENSG00000164045 | -2.87 | 1.60E-16 |
|  | *MAD2L1* | ENSG00000164109 | -2.84 | 1.22E-14 |
|  | *ANAPC13* | ENSG00000129055 | -2.79 | 1.99E-12 |
|  | *MDM2* | ENSG00000135679 | -2.76 | 1.02E-13 |
|  | *BCCIP* | ENSG00000107949 | -2.75 | 2.29E-11 |
|  | *MPHOSPH10* | ENSG00000124383 | -2.74 | 2.59E-12 |
|  | *BUB1* | ENSG00000169679 | -2.74 | 6.33E-15 |
|  | *KNSTRN* | ENSG00000128944 | -2.73 | 1.49E-13 |
|  | *ASPM* | ENSG00000066279 | -2.68 | 4.88E-13 |
|  | *SPC24* | ENSG00000161888 | -2.68 | 7.26E-14 |
|  | *INCENP* | ENSG00000149503 | -2.66 | 5.34E-17 |
|  | *NDC80* | ENSG00000080986 | -2.64 | 7.50E-11 |
|  | *MCM10* | ENSG00000065328 | -2.59 | 5.26E-13 |
|  | *NUP107* | ENSG00000111581 | -2.57 | 4.56E-11 |
|  | *ANAPC7* | ENSG00000196510 | -2.55 | 2.49E-12 |
|  | *E2F1* | ENSG00000101412 | -2.55 | 1.62E-13 |
|  | *KNL1* | ENSG00000137812 | -2.55 | 4.72E-11 |
|  | *MPHOSPH6* | ENSG00000135698 | -2.54 | 9.99E-12 |
|  | *NET1* | ENSG00000173848 | -2.53 | 5.92E-13 |
|  | *RFC5* | ENSG00000111445 | -2.51 | 4.56E-11 |
|  | *CENPM* | ENSG00000100162 | -2.51 | 1.31E-11 |
|  | *PALB2* | ENSG00000083093 | -2.46 | 4.70E-11 |
|  | *PLK3* | ENSG00000173846 | -2.43 | 2.28E-10 |
|  | *TICRR* | ENSG00000140534 | -2.41 | 4.60E-10 |
|  | *SKA3* | ENSG00000165480 | -2.38 | 1.04E-10 |
|  | *RFC2* | ENSG00000049541 | -2.37 | 3.86E-12 |
|  | *ESPL1* | ENSG00000135476 | -2.36 | 9.14E-13 |
|  | *BUB1B* | ENSG00000156970 | -2.34 | 3.03E-09 |
|  | *RFC4* | ENSG00000163918 | -2.33 | 1.97E-09 |
|  | *CENPJ* | ENSG00000151849 | -2.33 | 3.61E-10 |
|  | *CDT1* | ENSG00000167513 | -2.33 | 4.70E-11 |
|  | *PIMREG* | ENSG00000129195 | -2.31 | 8.64E-11 |
|  | *NUF2* | ENSG00000143228 | -2.31 | 1.76E-08 |
|  | *DSN1* | ENSG00000149636 | -2.30 | 1.75E-09 |
|  | *ESCO2* | ENSG00000171320 | -2.29 | 9.90E-10 |
|  | *CCSAP* | ENSG00000154429 | -2.28 | 2.99E-10 |
|  | *CENPF* | ENSG00000117724 | -2.28 | 1.64E-09 |
|  | *RPA2* | ENSG00000117748 | -2.27 | 4.48E-10 |
|  | *SPDL1* | ENSG00000040275 | -2.25 | 6.12E-10 |
|  | *CENPU* | ENSG00000151725 | -2.24 | 8.48E-09 |
|  | *MZT1* | ENSG00000204899 | -2.22 | 3.40E-10 |
|  | *E2F4* | ENSG00000205250 | -2.21 | 5.29E-11 |
|  | *SHCBP1* | ENSG00000171241 | -2.20 | 8.85E-10 |
|  | *CDK1* | ENSG00000170312 | -2.19 | 2.14E-07 |
|  | *ANAPC1* | ENSG00000153107 | -2.18 | 1.45E-09 |
|  | *BUB3* | ENSG00000154473 | -2.17 | 3.72E-10 |
|  | *MIS12* | ENSG00000167842 | -2.17 | 3.78E-08 |
|  | *PDS5A* | ENSG00000121892 | -2.17 | 4.72E-10 |
|  | *CNTROB* | ENSG00000170037 | -2.16 | 1.15E-09 |
|  | *CENPW* | ENSG00000203760 | -2.11 | 4.11E-08 |
|  | *ZWILCH* | ENSG00000174442 | -2.10 | 5.10E-08 |
|  | *CENPA* | ENSG00000115163 | -2.10 | 5.71E-10 |
|  | *ANAPC15* | ENSG00000110200 | -2.09 | 3.59E-06 |
|  | *SKA1* | ENSG00000154839 | -2.09 | 1.15E-08 |
|  | *CCNE1* | ENSG00000105173 | -2.09 | 6.39E-08 |
|  | *MZT2B* | ENSG00000152082 | -2.08 | 1.42E-08 |
|  | *DNA2* | ENSG00000138346 | -2.07 | 1.60E-08 |
|  | *SMU1* | ENSG00000122692 | -2.06 | 4.93E-08 |
|  | *WAPL* | ENSG00000062650 | -2.06 | 6.92E-09 |
|  | *MPHOSPH8* | ENSG00000196199 | -2.04 | 1.93E-08 |
|  | *NUSAP1* | ENSG00000137804 | -2.03 | 1.94E-07 |
|  | *BAP1* | ENSG00000163930 | -2.01 | 4.67E-10 |
|  | *ZW10* | ENSG00000086827 | -2.01 | 7.14E-08 |
|  | *NOC3L* | ENSG00000173145 | -2.01 | 1.61E-06 |
|  | *RAD21* | ENSG00000164754 | -1.98 | 1.25E-08 |
|  | *KNTC1* | ENSG00000184445 | -1.96 | 1.250E-07 |
|  | *RFC1* | ENSG00000035928 | -1.96 | 3.41E-07 |
|  | *BRCA2* | ENSG00000139618 | -1.94 | 8.48E-08 |
|  | *PRCC* | ENSG00000143294 | -1.93 | 9.86E-10 |
|  | *MAU2* | ENSG00000129933 | -1.92 | 2.76E-09 |
|  | *BRAP* | ENSG00000089234 | -1.91 | 1.57E-07 |
|  | *MOB1A* | ENSG00000114978 | -1.91 | 2.28E-08 |
|  | *CENPH* | ENSG00000153044 | -1.91 | 4.68E-07 |
|  | *MPHOSPH9* | ENSG00000051825 | -1.90 | 2.65E-07 |
|  | *RPA1* | ENSG00000132383 | -1.89 | 9.55E-10 |
|  | *ANAPC5* | ENSG00000089053 | -1.89 | 5.34E-08 |
|  | *E2F2* | ENSG00000007968 | -1.89 | 1.51E-08 |
|  | *CENPQ* | ENSG00000031691 | -1.88 | 3.92E-06 |
|  | *BRCA1* | ENSG00000012048 | -1.86 | 1.32E-07 |
|  | *MPLKIP* | ENSG00000168303 | -1.85 | 3.80E-07 |
|  | *AC083899.1* | ENSG00000204745 | -1.83 | 7.22E-07 |
|  | *CDK10* | ENSG00000185324 | -1.83 | 5.38E-07 |
|  | *CENPK* | ENSG00000123219 | -1.82 | 1.12E-05 |
|  | *CENPO* | ENSG00000138092 | -1.79 | 1.17E-06 |
|  | *MAD2L2* | ENSG00000116670 | -1.76 | 1.50E-05 |
|  | *ATM* | ENSG00000149311 | -1.76 | 2.04E-06 |
|  | *PLK2* | ENSG00000145632 | -1.75 | 5.31E-07 |
|  | *CDK2AP1* | ENSG00000111328 | -1.74 | 2.03E-08 |
|  | *RFC3* | ENSG00000133119 | -1.74 | 6.95E-07 |
|  | *SKA2* | ENSG00000182628 | -1.71 | 6.76E-06 |
|  | *ANAPC2* | ENSG00000176248 | -1.69 | 6.74E-06 |
|  | *RTF2* | ENSG00000022277 | -1.66 | 1.24E-05 |
|  | *CENPC* | ENSG00000145241 | -1.66 | 9.53E-06 |
|  | *ERICD* | ENSG00000280303 | -1.65 | 0.0314 |
|  | *SUGT1* | ENSG00000165416 | -1.64 | 8.44E-06 |
|  | *GSPT2* | ENSG00000189369 | -1.64 | 7.33E-05 |
|  | *ANAPC4* | ENSG00000053900 | -1.64 | 1.28E-05 |
|  | *ESCO1* | ENSG00000141446 | -1.63 | 3.03E-05 |
|  | *AC125232.1* | ENSG00000231259 | -1.62 | 0.000244 |
|  | *ANAPC16* | ENSG00000166295 | -1.60 | 1.59E-05 |
|  | *EAPP* | ENSG00000129518 | -1.60 | 6.02E-05 |
|  | *DSCC1* | ENSG00000136982 | -1.60 | 7.06E-05 |
|  | *ANAPC11* | ENSG00000141552 | -1.59 | 1.28E-05 |
|  | *CENPL* | ENSG00000120334 | -1.58 | 2.89E-05 |
|  | *BARD1* | ENSG00000138376 | -1.58 | 5.25E-06 |
|  | *E2F3* | ENSG00000112242 | -1.56 | 8.86E-07 |
|  | *PDS5B* | ENSG00000083642 | -1.55 | 6.76E-06 |
|  | *CDK7* | ENSG00000134058 | -1.55 | 7.33E-05 |
|  | *MZT2A* | ENSG00000173272 | -1.55 | 2.33E-05 |
|  | *BRAT1* | ENSG00000106009 | -1.53 | 4.64E-06 |
|  | *SPC25* | ENSG00000152253 | -1.46 | 1.59E-05 |
|  | *SKP1* | ENSG00000113558 | -1.45 | 5.73E-05 |
|  | *GAK* | ENSG00000178950 | -1.43 | 2.28E-05 |
|  | *CCNB1IP1* | ENSG00000100814 | -1.38 | 0.000220 |
|  | *MTBP* | ENSG00000172167 | -1.33 | 0.000510 |
|  | *CSPP1* | ENSG00000104218 | -1.33 | 0.000210 |
|  | *SFRP1* | ENSG00000104332 | -1.33 | 6.54E-05 |
|  | *NSL1* | ENSG00000117697 | -1.33 | 0.000230 |
|  | *G2E3* | ENSG00000092140 | -1.32 | 0.000370 |
|  | *NIPBL* | ENSG00000164190 | -1.31 | 7.25E-05 |
|  | *SWI5* | ENSG00000175854 | -1.28 | 0.00118 |
|  | *CDKN2C* | ENSG00000123080 | -1.26 | 0.00223 |
|  | *INCA1* | ENSG00000196388 | -1.25 | 0.00493 |
|  | *NUMA1* | ENSG00000137497 | -1.19 | 0.000210 |
|  | *SPICE1* | ENSG00000163611 | -1.14 | 0.00450 |
|  | *BRIP1* | ENSG00000136492 | -1.05 | 0.00359 |
|  | *CDK6* | ENSG00000105810 | -1.00 | 0.00190 |
|  | *CTC1* | ENSG00000178971 | -0.97 | 0.00855 |
|  | *CENPI* | ENSG00000102384 | -0.97 | 0.00792 |
|  | *MOB1B* | ENSG00000173542 | -0.89 | 0.0101 |
|  | *NPDC1* | ENSG00000107281 | -0.88 | 0.0318 |
|  | *MAD1L1* | ENSG00000002822 | -0.81 | 0.0148 |
|  | *BTG4* | ENSG00000137707 | 0.91 | 0.0268 |
|  | *SAMSN1* | ENSG00000155307 | 1.21 | 0.00212 |
|  | *RAD21L1* | ENSG00000244588 | 1.29 | 0.0264 |
|  | *CCNA1* | ENSG00000133101 | 1.46 | 0.0448 |
| Neurons | *GAP43* | ENSG00000172020 | 0.89 | 0.0143 |
|  | *NRXN1* | ENSG00000179915 | 0.90 | 0.0250 |
|  | *MAPT* | ENSG00000186868 | 0.98 | 0.00922 |
|  | *NRXN2* | ENSG00000110076 | 0.98 | 0.00815 |
|  | *NAV1* | ENSG00000134369 | 1.00 | 0.0103 |
|  | *SYT6* | ENSG00000134207 | 1.01 | 0.00975 |
|  | *SYNPO* | ENSG00000171992 | 1.05 | 0.00389 |
|  | *SYNPO2* | ENSG00000172403 | 1.16 | 0.000840 |
|  | *SHANK2* | ENSG00000162105 | 1.17 | 0.00106 |
|  | *NAV3* | ENSG00000067798 | 1.18 | 0.000838 |
|  | *NEXMIF* | ENSG00000050030 | 1.19 | 0.000838 |
|  | *SV2B* | ENSG00000185518 | 1.23 | 0.000633 |
|  | *SYT2* | ENSG00000143858 | 1.23 | 0.00140 |
|  | *SYNPR* | ENSG00000163630 | 1.27 | 0.000453 |
|  | *PSD-93* | ENSG00000150672 | 1.27 | 0.000465 |
|  | *SV2C* | ENSG00000122012 | 1.29 | 0.000276 |
|  | *NELL2* | ENSG00000184613 | 1.34 | 0.000277 |
|  | *SYN3* | ENSG00000185666 | 1.35 | 0.000266 |
|  | *SYT9* | ENSG00000170743 | 1.36 | 0.000173 |
|  | *CDH12* | ENSG00000154162 | 1.37 | 0.000515 |
|  | *NELL1* | ENSG00000165973 | 1.38 | 0.000183 |
|  | *SNAP91* | ENSG00000065609 | 1.41 | 0.000680 |
|  | *NEGR1* | ENSG00000172260 | 1.41 | 0.000328 |
|  | *SYT16* | ENSG00000139973 | 1.43 | 9.69E-05 |
|  | *NRG1* | ENSG00000157168 | 1.44 | 0.000154 |
|  | *NRXN3* | ENSG00000021645 | 1.46 | 0.000119 |
|  | *ELAVL4* | ENSG00000162374 | 1.57 | 9.45E-05 |
|  | *SYT13* | ENSG00000019505 | 1.66 | 0.000346 |
|  | *SYT10* | ENSG00000110975 | 1.77 | 0.000396 |
|  | *NPY* | ENSG00000122585 | 2.29 | 0.0186 |
|  | *SYT4* | ENSG00000132872 | 2.75 | 2.36E-07 |
|  | *APOE* | ENSG00000130203 | 2.92 | 7.57E-13 |
| Neural plasticity | *PLXNA1* | ENSG00000114554 | -1.96 | 2.82E-10 |
|  | *SPOCK2* | ENSG00000107742 | -1.93 | 3.48E-10 |
|  | *PTENP1* | ENSG00000237984 | -1.69 | 0.00264 |
|  | *PTEN* | ENSG00000171862 | -1.60 | 8.11E-06 |
|  | *SEMA3A* | ENSG00000075213 | -1.54 | 7.88E-06 |
|  | *SEMA3E* | ENSG00000170381 | -1.43 | 5.22E-05 |
|  | *SNCAIP* | ENSG00000064692 | -0.91 | 0.0144 |
|  | *ULK4* | ENSG00000168038 | 0.65 | 0.0433 |
|  | *SPOCK1* | ENSG00000152377 | 0.70 | 0.0349 |
|  | *SEMA3D* | ENSG00000153993 | 0.99 | 0.00812 |
|  | *NAV1* | ENSG00000134369 | 0.10 | 0.0103 |
|  | *SYNPO* | ENSG00000171992 | 1.05 | 0.00389 |
|  | *SRGAP1* | ENSG00000196935 | 1.17 | 0.00548 |
|  | *NAV3* | ENSG00000067798 | 1.18 | 0.000838 |
|  | *UNC5C* | ENSG00000182168 | 1.19 | 0.000796 |
|  | *RASGRF2* | ENSG00000113319 | 1.20 | 0.00309 |
|  | *ASTN2* | ENSG00000148219 | 1.25 | 0.000420 |
|  | *SEMA3C* | ENSG00000075223 | 1.27 | 0.00116 |
|  | *STMN2* | ENSG00000104435 | 1.28 | 0.00711 |
|  | *TENM2* | ENSG00000145934 | 1.28 | 0.000423 |
|  | *DCC* | ENSG00000187323 | 1.30 | 0.000329 |
|  | *ASTN1* | ENSG00000152092 | 1.31 | 0.000670 |
|  | *SLIT3* | ENSG00000184347 | 1.37 | 0.000459 |
|  | *SPOCK3* | ENSG00000196104 | 1.42 | 0.000760 |
|  | *SLITRK6* | ENSG00000184564 | 1.73 | 0.0159 |
|  | *UNCX* | ENSG00000164853 | 3.09 | 0.0350 |
| NTFs and their receptors | *FGF18* | ENSG00000156427 | 1.07 | 0.0328 |
|  | *FGF10* | ENSG00000070193 | 1.19 | 0.00779 |
|  | *FGF13* | ENSG00000129682 | 1.19 | 0.000565 |
|  | *FGF12* | ENSG00000114279 | 1.25 | 0.000510 |
|  | *FGF5* | ENSG00000138675 | 1.38 | 0.00949 |
|  | *NDNF* | ENSG00000173376 | 1.48 | 0.00201 |
|  | *FGF23* | ENSG00000118972 | 1.60 | 0.0148 |
| Astrocytes | *BIRC5* | ENSG00000089685 | -2.30 | 5.95E-11 |
|  | *GJA1* | ENSG00000152661 | -1.29 | 0.000957 |
|  | *GAP43* | ENSG00000172020 | 0.89 | 0.0143 |
|  | *NDRG2* | ENSG00000165795 | 0.89 | 0.0237 |
| Oligodendrocytes | *ERBB3* | ENSG00000065361 | 0.98 | 0.0471 |
|  | *NFASC* | ENSG00000163531 | 1.24 | 0.00125 |
|  | *PLP1* | ENSG00000123560 | 1.28 | 0.00462 |
|  | *MYT1* | ENSG00000196132 | 1.31 | 0.000399 |
|  | *CNTNAP2* | ENSG00000174469 | 1.37 | 0.000296 |
|  | *NRG1* | ENSG00000157168 | 1.44 | 0.000154 |
| Serotonergic neurons | *HMCES* | ENSG00000183624 | -1.93 | 1.36E-08 |
|  | *HTR7* | ENSG00000148680 | 0.92 | 0.00936 |
|  | *HTR5BP* | ENSG00000125631 | 0.99 | 0.0274 |
|  | *HTR2C* | ENSG00000147246 | 1.23 | 0.00434 |
|  | *HTR3B* | ENSG00000149305 | 1.24 | 0.00501 |
|  | *HTR1E* | ENSG00000168830 | 1.24 | 0.00140 |
|  | *HTR2A* | ENSG00000102468 | 1.25 | 0.00252 |
|  | *HTR5A* | ENSG00000157219 | 1.26 | 0.0339 |
|  | *HTR4* | ENSG00000164270 | 1.38 | 0.000280 |
|  | *DDC* | ENSG00000132437 | 1.53 | 0.00556 |
|  | *HTR6* | ENSG00000158748 | 1.67 | 0.0143 |
|  | *HTR1F* | ENSG00000179097 | 2.27 | 0.0356 |
| GABAergic neurons | *GABRG2* | ENSG00000113327 | 1.14 | 0.00235 |
|  | *SLC6A13* | ENSG00000010379 | 1.14 | 0.0289 |
|  | *GABRA6* | ENSG00000145863 | 1.16 | 0.00215 |
|  | *GABRA2* | ENSG00000151834 | 1.17 | 0.00178 |
|  | *GABRR3* | ENSG00000183185 | 1.18 | 0.00629 |
|  | *GABRG1* | ENSG00000163285 | 1.21 | 0.00305 |
|  | *GAD1* | ENSG00000128683 | 1.22 | 0.00578 |
|  | *GABRB3* | ENSG00000166206 | 1.25 | 0.00024 |
|  | *SLC6A12* | ENSG00000111181 | 1.27 | 0.0263 |
|  | *GABRQ* | ENSG00000268089 | 1.27 | 0.0114 |
|  | *GABRG3* | ENSG00000182256 | 1.27 | 0.00039 |
|  | *GABRA1* | ENSG00000022355 | 1.33 | 0.00727 |
|  | *GABRB1* | ENSG00000163288 | 1.36 | 0.00023 |
|  | *GAD2* | ENSG00000136750 | 1.38 | 0.00396 |
|  | *GABRR1* | ENSG00000146276 | 1.39 | 0.00738 |
|  | *GABRP* | ENSG00000094755 | 1.40 | 0.0123 |
|  | *GABRA3* | ENSG00000011677 | 1.42 | 0.000305 |
|  | *GABRB2* | ENSG00000145864 | 1.50 | 3.36E-05 |
|  | *GABRA4* | ENSG00000109158 | 1.54 | 0.000342 |
| Glutamatergic neurons | *SLC1A3* | ENSG00000079215 | -1.65 | 5.29E-06 |
|  | *SLC17A5* | ENSG00000119899 | -1.11 | 0.00152 |
|  | *GRIP2* | ENSG00000144596 | -1.03 | 0.00359 |
|  | *GRIK5* | ENSG00000105737 | 0.85 | 0.0446 |
|  | *GRIN3A* | ENSG00000198785 | 1.11 | 0.00241 |
|  | *SLC1A1* | ENSG00000106688 | 1.19 | 0.00230 |
|  | *GRIA2* | ENSG00000120251 | 1.19 | 0.000862 |
|  | *SLC17A4* | ENSG00000146039 | 1.24 | 0.00784 |
|  | *GRIK1* | ENSG00000171189 | 1.26 | 0.00106 |
|  | *GRID1* | ENSG00000182771 | 1.28 | 0.000107 |
|  | *SLC17A3* | ENSG00000124564 | 1.28 | 0.00229 |
|  | *GRIK4* | ENSG00000149403 | 1.31 | 0.000520 |
|  | *GRM8* | ENSG00000179603 | 1.33 | 0.000194 |
|  | *GRIK3* | ENSG00000163873 | 1.35 | 0.000180 |
|  | *GRIK2* | ENSG00000164418 | 1.37 | 0.000320 |
|  | *GRID2* | ENSG00000152208 | 1.39 | 0.000176 |
|  | *GRM5* | ENSG00000168959 | 1.40 | 0.000299 |
|  | *GRM1* | ENSG00000152822 | 1.40 | 0.000283 |
|  | *GRM7* | ENSG00000196277 | 1.45 | 9.91E-05 |
|  | *SLC17A8* | ENSG00000179520 | 1.47 | 0.000680 |
|  | *GRIN2A* | ENSG00000183454 | 1.53 | 5.24E-05 |
|  | *GRM3* | ENSG00000198822 | 1.57 | 6.06E-05 |
| Cholinergic neurons | *CHRNA6* | ENSG00000147434 | 1.28 | 0.0101 |
|  | *CHRNA1* | ENSG00000138435 | 1.37 | 0.0264 |
|  | *CHRNB4* | ENSG00000117971 | 1.51 | 0.00121 |
|  | *CHRNA2* | ENSG00000120903 | 1.67 | 0.0157 |
|  | *SLC5A7* | ENSG00000115665 | 1.78 | 0.00633 |
|  | *CHRND* | ENSG00000135902 | 1.88 | 0.00308 |
|  | *CHRNA9* | ENSG00000174343 | 2.58 | 1.16E-09 |
| Familial PD | *SNCA* | ENSG00000145335 | 1.27 | 0.00360 |
|  | *LRRK2* | ENSG00000188906 | 1.30 | 0.000569 |

**Supplementary Table S3.** List of genes differentially expressed by differentiated hVM1 clone 32 cells. Genes are categorized by their association with cancer, glucose metabolism, energy metabolism, lipid metabolism, and calcium signaling, in addition to a category for other genes of interest.

| **Category** | **Gene symbol** | **Gene ID** | **Fold change** | **q value** |
| --- | --- | --- | --- | --- |
| Cancer | *CASC2* | ENSG00000177640 | 0.93 | 0.00685 |
|  | *CASC18* | ENSG00000257859 | 1.06 | 0.0180 |
|  | *CASC1* | ENSG00000118307 | 1.08 | 0.00418 |
|  | *CASC16* | ENSG00000249231 | 1.16 | 0.00168 |
|  | *CASC9* | ENSG00000249395 | 1.16 | 0.00646 |
|  | *CASC8* | ENSG00000246228 | 1.26 | 0.00130 |
|  | *CASC11* | ENSG00000249375 | 1.31 | 0.00216 |
|  | *CASC20* | ENSG00000229876 | 1.48 | 8.82E-05 |
|  | *CASC6* | ENSG00000224944 | 1.56 | 9.79E-05 |
|  | *CASC17* | ENSG00000260785 | 1.70 | 0.000144 |
|  | *CASC23* | ENSG00000255420 | 2.30 | 0.0173 |
| Glucose metabolism | *INSL6* | ENSG00000120210 | 0.99 | 0.0102 |
|  | *IGF1* | ENSG00000017427 | 1.28 | 0.0328 |
|  | *IGFBP7* | ENSG00000163453 | 1.29 | 0.000634 |
|  | *GLP2R* | ENSG00000065325 | 1.30 | 0.00139 |
|  | *INSL5* | ENSG00000172410 | 2.75 | 0.0428 |
| Energy metabolism | *ABCG1* | ENSG00000160179 | 0.85 | 0.0401 |
|  | *ABCC3* | ENSG00000108846 | 0.89 | 0.0263 |
|  | *ABCC8* | ENSG00000006071 | 1.01 | 0.0190 |
|  | *ABCA12* | ENSG00000144452 | 1.04 | 0.00603 |
|  | *ABCG8* | ENSG00000143921 | 1.06 | 0.00823 |
|  | *ABCC12* | ENSG00000140798 | 1.07 | 0.00830 |
|  | *ABCC6* | ENSG00000091262 | 1.08 | 0.00874 |
|  | *ABCG5* | ENSG00000138075 | 1.10 | 0.0368 |
|  | *MARC2* | ENSG00000117791 | 1.14 | 0.00476 |
|  | *ABCB1* | ENSG00000085563 | 1.16 | 0.00859 |
|  | *ABCA13* | ENSG00000179869 | 1.17 | 0.00105 |
|  | *ABCA6* | ENSG00000154262 | 1.18 | 0.00728 |
|  | *ABCC11* | ENSG00000121270 | 1.19 | 0.00355 |
|  | *ABCB11* | ENSG00000073734 | 1.37 | 0.000665 |
|  | *ABCB4* | ENSG00000005471 | 1.41 | 0.00134 |
|  | *ABCA9* | ENSG00000154258 | 1.45 | 0.0102 |
|  | *ABCD2* | ENSG00000173208 | 1.49 | 9.67E-05 |
|  | *ABCB5* | ENSG00000004846 | 1.53 | 0.000206 |
|  | *ABCA8* | ENSG00000141338 | 2.37 | 4.60E-11 |
| Lipid metabolism | *PLD1* | ENSG00000075651 | 0.76 | 0.0247 |
|  | *ATP8A1* | ENSG00000124406 | 0.85 | 0.0108 |
|  | *PLCB4* | ENSG00000101333 | 0.91 | 0.00491 |
|  | *MGLL* | ENSG00000074416 | 0.93 | 0.0137 |
|  | *FABP6* | ENSG00000170231 | 0.97 | 0.0417 |
|  | *ACOT11* | ENSG00000162390 | 1.00 | 0.0384 |
|  | *FAAH2* | ENSG00000165591 | 1.01 | 0.00855 |
| **Category** | **Gene symbol** | **Gene ID** | **Fold change** | **q value** |
|  | *ATP8B1* | ENSG00000081923 | 1.02 | 0.0145 |
|  | *ACSBG1* | ENSG00000103740 | 1.02 | 0.0113 |
|  | *LIPK* | ENSG00000204021 | 1.05 | 0.0325 |
|  | *ACOT12* | ENSG00000172497 | 1.06 | 0.00735 |
|  | *FFAR4* | ENSG00000186188 | 1.07 | 0.0367 |
|  | *ACOX2* | ENSG00000168306 | 1.10 | 0.0103 |
|  | *ELOVL7* | ENSG00000164181 | 1.11 | 0.00921 |
|  | *HMGCS2* | ENSG00000134240 | 1.14 | 0.0230 |
|  | *ACSM2B* | ENSG00000066813 | 1.14 | 0.0156 |
|  | *ACSM4* | ENSG00000215009 | 1.18 | 0.0262 |
|  | *PLB1* | ENSG00000163803 | 1.20 | 0.00160 |
|  | *ACSM2A* | ENSG00000183747 | 1.23 | 0.0128 |
|  | *PLA2G4A* | ENSG00000116711 | 1.27 | 0.00160 |
|  | *PLP1* | ENSG00000123560 | 1.28 | 0.00462 |
|  | *ATP8A2* | ENSG00000132932 | 1.30 | 0.000261 |
|  | *PLA2G7* | ENSG00000146070 | 1.35 | 0.00383 |
|  | *PLCG2* | ENSG00000197943 | 1.35 | 0.000416 |
|  | *LIPJ* | ENSG00000204022 | 1.37 | 0.0118 |
|  | *LPA* | ENSG00000198670 | 1.37 | 0.000644 |
|  | *PLPP4* | ENSG00000203805 | 1.40 | 0.00132 |
|  | *PLD5* | ENSG00000180287 | 1.40 | 0.000280 |
|  | *PHYHIP* | ENSG00000168490 | 1.42 | 0.00189 |
|  | *PLA2G5* | ENSG00000127472 | 1.46 | 0.000151 |
|  | *ACSL5* | ENSG00000197142 | 1.48 | 0.0136 |
|  | *PLA2G4D* | ENSG00000159337 | 1.62 | 0.00335 |
|  | *ACSM5* | ENSG00000183549 | 1.66 | 0.00249 |
|  | *PLA2G4F* | ENSG00000168907 | 1.66 | 0.00704 |
|  | *LIPN* | ENSG00000204020 | 1.67 | 0.0257 |
|  | *PLA2G12B* | ENSG00000138308 | 1.69 | 0.00281 |
|  | *PLA1A* | ENSG00000144837 | 1.74 | 0.000323 |
|  | *LIPM* | ENSG00000173239 | 1.89 | 0.000489 |
| Calcium signaling | *CACNA2D1* | ENSG00000153956 | 0.69 | 0.0380 |
|  | *CACNA1G* | ENSG00000006283 | 0.93 | 0.0183 |
|  | *CAMK2A* | ENSG00000070808 | 0.96 | 0.0137 |
|  | *CACNG2* | ENSG00000166862 | 0.97 | 0.00882 |
|  | *SMOC2* | ENSG00000112562 | 1.08 | 0.00236 |
|  | *C2CD6* | ENSG00000155754 | 1.08 | 0.00549 |
|  | *CACNA2D4* | ENSG00000151062 | 1.10 | 0.00339 |
|  | *CACNA1B* | ENSG00000148408 | 1.10 | 0.00127 |
|  | *CAMK4* | ENSG00000152495 | 1.11 | 0.00224 |
|  | *ATP2C2* | ENSG00000064270 | 1.11 | 0.00489 |
|  | *CAMK1G* | ENSG00000008118 | 1.11 | 0.0128 |
|  | *CACNA1I* | ENSG00000100346 | 1.12 | 0.00912 |
|  | *ATP2A3* | ENSG00000074370 | 1.12 | 0.0263 |
| **Category** | **Gene symbol** | **Gene ID** | **Fold change** | **q value** |
|  | *CACNG3* | ENSG00000006116 | 1.12 | 0.00183 |
|  | *CIB4* | ENSG00000157884 | 1.13 | 0.00591 |
|  | *CACNA2D2* | ENSG00000007402 | 1.15 | 0.00717 |
|  | *CACNG4* | ENSG00000075461 | 1.16 | 0.00213 |
|  | *CACNA1D* | ENSG00000157388 | 1.17 | 0.00101 |
|  | *CASR* | ENSG00000036828 | 1.21 | 0.00124 |
|  | *CACNB2* | ENSG00000165995 | 1.23 | 0.000699 |
|  | *CACNA1S* | ENSG00000081248 | 1.27 | 0.00837 |
|  | *CACNA1F* | ENSG00000102001 | 1.27 | 0.0155 |
|  | *CACNA1E* | ENSG00000198216 | 1.29 | 0.000308 |
|  | *CACNA1C* | ENSG00000151067 | 1.31 | 0.000586 |
|  | *CACNB4* | ENSG00000182389 | 1.33 | 0.000193 |
|  | *SCGN* | ENSG00000079689 | 1.33 | 0.00124 |
|  | *CACNA1A* | ENSG00000141837 | 1.35 | 0.000285 |
|  | *CACNA2D3* | ENSG00000157445 | 1.37 | 0.000123 |
|  | *ATP2B3* | ENSG00000067842 | 1.42 | 0.00248 |
|  | *CACNG7* | ENSG00000105605 | 1.52 | 0.00173 |
|  | *CACNG5* | ENSG00000075429 | 1.57 | 1.87E-05 |
|  | *CACNA1H* | ENSG00000196557 | 1.69 | 1.39E-05 |
|  | *CACNG1* | ENSG00000108878 | 1.72 | 0.00913 |
|  | *CALHM4* | ENSG00000164451 | 2.07 | 0.00288 |
| Other | *MAPK4* | ENSG00000141639 | 1.13 | 0.00225 |
|  | *PGR* | ENSG00000082175 | 1.14 | 0.00523 |
|  | *PPARG* | ENSG00000132170 | 1.23 | 0.000640 |
|  | *HDAC9* | ENSG00000048052 | 1.30 | 0.000572 |
|  | *PPARGC1A* | ENSG00000109819 | 1.52 | 4.88E-05 |
|  | *PDGFD* | ENSG00000170962 | 1.52 | 5.19E-05 |
